# Targeted inhibition of protein synthesis renders cancer cells vulnerable to apoptosis by unfolded protein response

**DOI:** 10.1038/s41419-023-06055-w

**Published:** 2023-08-26

**Authors:** Franziska Gsottberger, Christina Meier, Anna Ammon, Scott Parker, Kerstin Wendland, Rebekka George, Srdjan Petkovic, Lisa Mellenthin, Charlotte Emmerich, Gloria Lutzny-Geier, Markus Metzler, Andreas Mackensen, Vidyalakshmi Chandramohan, Fabian Müller

**Affiliations:** 1 Department of Internal Medicine 5, Haematology and Oncology, University Hospital of Erlangen, Friedrich-Alexander University of Erlangen-Nuremberg (FAU), Erlangen, Germany; 2 Department of Neurosurgery, Duke University Medical Center, Durham, NC USA; 3 Deptartment of Pediatrics and Adolescent Medicine, University Hospital of Erlangen, Friedrich-Alexander University of Erlangen-Nuremberg (FAU), Erlangen, Germany; 4 Bavarian Cancer Research Center (BZKF), Erlangen, Germany

**Keywords:** Cancer, Apoptosis

## Abstract

Cellular stress responses including the unfolded protein response (UPR) decide over the fate of an individual cell to ensure survival of the entire organism. During physiologic UPR counter-regulation, protective proteins are upregulated to prevent cell death. A similar strategy induces resistance to UPR in cancer. Therefore, we hypothesized that blocking protein synthesis following induction of UPR substantially enhances drug-induced apoptosis of malignant cells. In line, upregulation of the chaperone BiP was prevented by simultaneous arrest of protein synthesis in B cell malignancies. Cytotoxicity by immunotoxins—approved inhibitors of protein synthesis—was synergistically enhanced in combination with UPR-inducers in seven distinct hematologic and three solid tumor entities in vitro. Synergistic cell death depended on mitochondrial outer membrane permeabilization via BAK/BAX, which correlated with synergistic, IRE1α-dependent reduction of BID, accompanied by an additive fall of MCL-1. The strong synergy was reproduced in vivo against xenograft mouse models of mantle cell lymphoma, Burkitt’s lymphoma, and patient-derived acute lymphoblastic leukemia. In contrast, synergy was absent in blood cells of healthy donors suggesting a tumor-specific vulnerability. Together, these data support clinical evaluation of blocking stress response counter-regulation using inhibitors of protein synthesis as a novel therapeutic strategy.

## Introduction

Any cell that is exposed to cytotoxic conditions produces a stress response, which aims to repair damage and to protect the entire organism. If the stress response is irreversible or cannot be resolved in a timely manner, a programmed cell death pathway is executed [1]. Central to restoring cell homeostasis are newly synthesized proteins [2]. Hence, we hypothesized that cytotoxicity of a drug that induces a stress response is enhanced by a second drug that blocks production of new proteins, which by itself is also cytotoxic to a cell.

A well-studied example of stress responses is the unfolded protein response (UPR). UPR is initiated by accumulation of misfolded proteins in the endoplasmic reticulum (ER) [3, 4]. Under ER stress, misfolded proteins are bound by the chaperone ‘binding-immunoglobulin protein’ [BiP, also glucose-regulated protein 78 (GRP78), or heat shock protein family A member 5 (HSPA5)], which then dissociates from the three ER receptors inositol-requiring protein 1α (IRE1α), protein kinase RNA-like ER kinase (PERK), and activating transcription factor 6 (ATF6) [5–8]. Liberated IRE1α and PERK auto-phosphorylate, and, together with cleaved ATF6, orchestrate UPR [3, 9]. To resolve accumulated misfolded proteins, protein load is reduced by a PERK-induced transient block of protein synthesis and by regulated IRE1α-dependent decay (RIDD) of RNAs, while selective gene expression is induced via the transcription factors X-box binding protein 1 s (XBP1s), ATF4, and ATF6 [10–14]. In addition to reduction of misfolded proteins, upregulation of BiP, which binds and turns off UPR receptors, is key to restoring homeostasis [4, 5, 15–17]. If ER stress cannot be resolved in time, mitochondrial apoptosis is triggered through changes in B cell lymphoma 2 (BCL-2) family proteins [4, 18, 19]. However, cancer cells are frequently resistant to UPR-induced cell death through upregulation of proteins that attenuate UPR, including chaperones [20–23]. In line with the initial goal of drug synergy, simultaneous arrest of protein synthesis may counteract resistance to UPR and thus sensitize cancer cells more than healthy tissue to the combination treatment.

For analysis of drug synergy, mechanistically distinct drugs with similar functional outcome are tested. UPR can be induced by tunicamycin (TM), which irreversibly blocks N-linked protein glycosylation in the ER [24], by 2-Deoxyglucose (2-DG), which competes with mannose and reversibly inhibits N-linked glycosylation [25], or by proteasome inhibitor Bortezomib (BTZ), which impairs protein degradation [26]. Because excess mannose reverts 2-DG-induced UPR, 2-DG is useful for mechanistic studies [27]. Inhibition of protein synthesis is achieved by puromycin, which induces premature termination of polypeptide synthesis and by cycloheximide (CHX), which blocks translocation of tRNAs within the ribosome [28]. While both drugs are not cell selective, target cell-specific arrest of protein synthesis is achieved by immunotoxins, which are fusion proteins of a targeting moiety and *Pseudomonas* exotoxin A (PE) [29]. The catalytically active domain of PE ADP-ribosylates and inactivates eukaryotic elongation factor 2 (EF2), which arrests elongation at the ribosome. Upon block of protein synthesis by PE, the anti-apoptotic induced myeloid leukemia cell differentiation protein (MCL-1) is rapidly degraded due to its PEST-sequence, which induces BAK-dependent mitochondrial apoptosis [30, 31].

Here, we establish the mechanism by which UPR-induced apoptosis is synergistically enhanced by arrest of protein synthesis in various cancer entities.

## Results

### Protein synthesis inhibition prevents upregulation of BiP and results in synergistic cell death

**Fig. 1:**
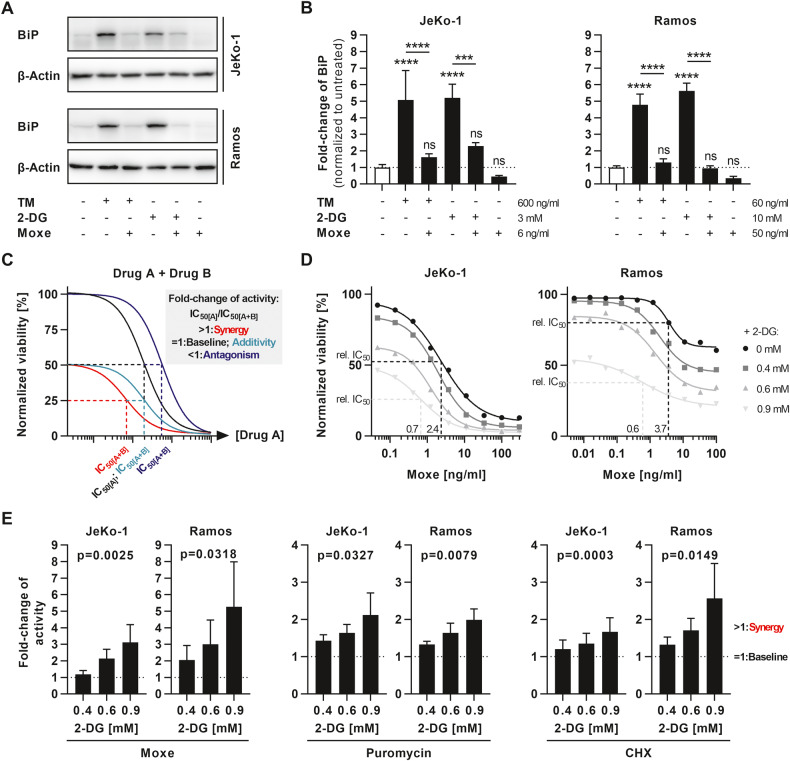
Combination of protein synthesis inhibition and UPR inducer 2-DG generates synergy by blocking UPR counter-regulation. **A**, **B** Cells were treated with tunicamycin (TM), 2-DG, and/or Moxe for 16 h. Protein levels of BiP and β-Actin were determined by western blot and quantified by densitometry. Bars show mean + SD of *n* = 4 replicates. *P*-values were determined by ordinary one-way ANOVA (Šídák’s test). **C** Exemplary dose-response curves of drug A (black) in combination with drug B and their respective IC_50,_ indicating synergistic (red), additive (turquoise), or antagonistic (blue) effects according to Bliss independence. **D**, **E** Cytotoxicity of Moxe (JeKo-1 *n* = 4, Ramos *n* = 3), puromycin (JeKo-1 *n* = 3, Ramos *n* = 3), or cycloheximide (CHX; JeKo-1 *n* = 5, Ramos *n* = 4) combined with 2-DG was determined by flow cytometry after 72 h. **D** shows representative dose-response curves normalized to untreated control including relative (rel.) IC_50_. **E** shows fold-change of drug activity and thus synergy over baseline of each drug alone as defined in (**C**). Bars show mean + SD normalized to the respective drug alone. *P*-values of linear trends were calculated by RM one-way ANOVA. *P*-values: not significant (ns): *p* > 0.05, ***p ≤ 0.001, ****p ≤ 0.0001 or as indicated.

Because upregulation of the chaperone BiP is central to restoring homeostasis after UPR, we initially tested whether an arrest of protein synthesis by the FDA-approved, CD22-targeted immunotoxin Moxetumomab pasudotox (Lumoxiti®, Moxe) influenced BiP expression in B cell malignancy cell lines JeKo-1 (mantle cell lymphoma, MCL) and Ramos (Burkitt’s lymphoma, BL) (Fig. 1A, B). In line with our hypothesis that arrest of protein synthesis blocks UPR counter-regulation, Moxe prevented upregulation of BiP after 2-DG and TM treatment in both cell lines. Next, we asked whether the combination resulted in synergistic cell death. To test for drug synergy, we analyzed the shift of dose-response curves of drug A by fixed concentrations of drug B and the respective IC_50_ values (Fig. 1C). According to Bliss independence [32], additivity of two drugs does not affect their IC_50_. Thus, the fold-change of activity, here defined as IC_50[A]_/IC_50[A+B]_, remains unchanged compared to baseline in case of additivity and equals 1, while values greater than 1 indicate synergy and values less than 1 indicate antagonism (Fig. 1C). In JeKo-1 and Ramos, 2-DG induced synergistic shifts of Moxe dose-response curves and the respective relative IC_50_, thus leading to a strong enhancement of cell killing in a concentration-dependent manner (Fig. 1D). The respective fold-change demonstrates that 2-DG not only synergistically enhanced activity of Moxe, but also increased activity of puromycin and of CHX in JeKo-1 and Ramos (Fig. 1E). Together, these data strongly suggest that 2-DG potentiates ribosome-targeting inhibitors of protein synthesis.

### Synergy is time-dependent, is mimicked by other UPR-inducers, and is reversed by mannose

**Fig. 2:**
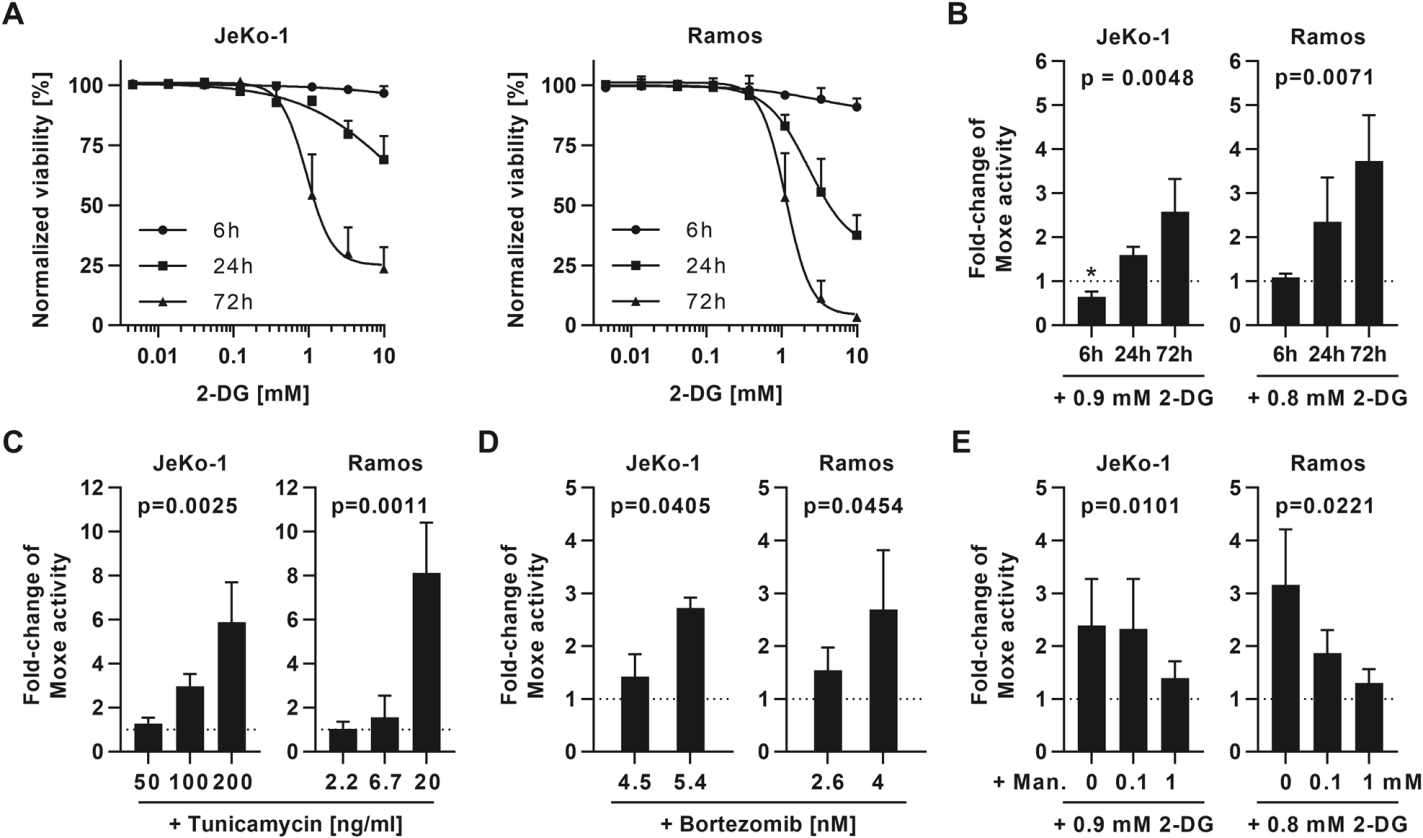
Synergy of Moxe and 2-DG is time-dependent, mimicked by TM and BTZ, and reversed by mannose. **A** Cells were treated with 2-DG for the indicated time and viability analyzed after 72 h by flow cytometry. Shown are 2-DG dose-response curves, normalized to untreated control, as mean + SD of *n* = 3 replicates. **B** Fold-change of Moxe activity (inverse IC_50_) by addition of 2-DG for the indicated time was measured after 72 h and normalized to Moxe alone. Bars represent mean + SD of *n* = 3 replicates. *P*-values of linear trends were calculated by RM one-way ANOVA. **C**, **D** Fold-change of Moxe activity by tunicamycin (TM; JeKo-1 *n* = 3, Ramos *n* = 4) or bortezomib (BTZ; JeKo-1 *n* = 3, Ramos *n* = 3) as mean + SD. *P*-values were calculated by paired *t*-test. **E** Fold-change of Moxe activity after addition of 2-DG and mannose (man.) at indicated concentrations (JeKo-1 *n* = 5, Ramos *n* = 3). Each bar represents mean + SD. *P*-values of linear trends by RM one-way ANOVA. Fold-change of activity >1 indicates synergy according to Bliss independence (**B**–**E**). *P*-values: *p ≤ 0.05, or as indicated.

Because cell death through UPR depends on a persisting stress signal, we tested for a time component of 2-DG-induced cell death and of the synergy. First, cells were treated with 2-DG for 6, 24, or 72 h, 2-DG was washed out, and cell viability was determined at 72 h (Fig. 2A). Viability of JeKo-1 and of Ramos decreased the longer 2-DG was present. Next, we treated cells continuously with Moxe for 72 h, while 2-DG exposure time varied (Fig. 2B). In both cell lines, increase in Moxe activity by 2-DG required an exposure time of at least 24 h. Thus, cytotoxicity of 2-DG and of the synergy of Moxe and 2-DG depended on persisting 2-DG exposure. In line with the above observation, Moxe in combination with UPR-inducers TM and BTZ showed a dose-dependent synergy against JeKo-1 and Ramos (Fig. 2C, D). Although 2-DG has other cellular effects, such as inhibition of glycolysis, we demonstrate that 2-DG-induced cell death in JeKo-1 and Ramos is efficiently reversed by addition of mannose, while pyruvate, which rescues ATP synthesis at a later enzymatic step of glycolysis, had little effect and ribose, which rescues the pentose phosphate pathway, had no effect on cell viability after 2-DG treatment (Fig. S1A). Moreover, only mannose, but not pyruvate nor ribose, reversed the synergy of Moxe and 2-DG in these cell lines in a dose-dependent manner (Fig. 2E and Fig. S1B). Together, these data strongly suggest that 2-DG-mediated cell death and synergy of 2-DG and Moxe are induced by a reversible inhibition of N-linked glycosylation and are connected with UPR.

### Synergistic cell death correlates with induction of UPR and mitochondrial apoptosis

**Fig. 3:**
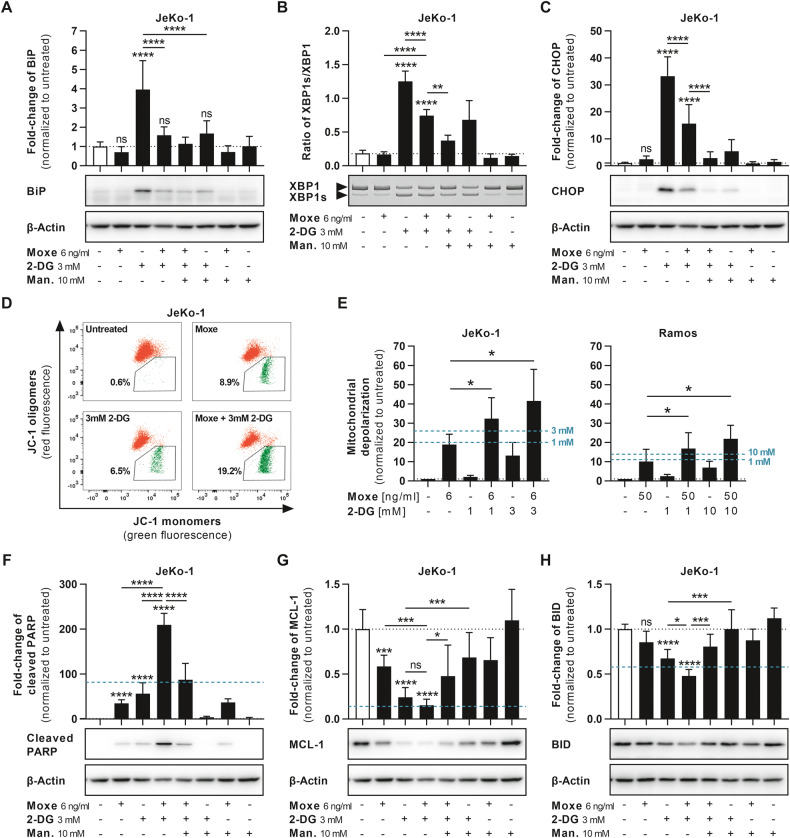
Combination of UPR and of protein synthesis inhibition synergistically induces apoptosis. **A**–**C** Cells were treated with Moxe, 2-DG, and mannose (man.) for 16 h. Protein levels of BiP (**A**; *n* = 6) and CHOP (**C**; *n* = 10) were analyzed by western blot using β-actin as control. Cleavage of XBP1 (**B**; *n* = 4) was analyzed by RT-PCR as ratio of XBP1s to XBP1 and quantified by densitometry. Each bar represents mean + SD. *P*-values were determined by ordinary one-way ANOVA (Šídák’s test). **D**, **E** Cells were treated with indicated drug concentrations and mitochondrial depolarization was determined as ratio of green to red emitting JC-1 by flow cytometry (JeKo-1 *n* = 5, Ramos *n* = 3). Each bar represents mean + SD. *P*-values were determined by RM one-way ANOVA (Dunnett’s test). **F**–**H** JeKo-1 were treated as in A and protein levels of cleaved PARP (**F**; *n* = 10), MCL-1 (**G**; *n* = 10), and BID (**H**; *n* = 6) were analyzed. Each bar represents mean + SD. *P*-values were determined by ordinary one-way ANOVA (Šídák’s test). Turquoise line indicates additive effects according to Bliss independence (**E**–**H**). *P*-values: not significant (ns): *p* > 0.05, *p ≤ 0.05, **p ≤ 0.01, ***p ≤ 0.001, ****p ≤ 0.0001.

To approach the underlying mechanism of drug synergy, we analyzed signaling pathways of cell death and of UPR. As discussed above, BiP was no longer significantly upregulated by 2-DG after the combination with Moxe or with mannose in JeKo-1 and Ramos (Fig. 3A and S2A). To test whether UPR-signaling remained active after Moxe and 2-DG, we determined cleavage of XBP1 on mRNA level as ratio of XBP1s (spliced) to XBP1 using RT-PCR (Fig. 3B and S2B). Although 2-DG-induced XBP1 cleavage was reduced in combination with Moxe, it remained significantly elevated compared to Moxe alone and was partially reversed by mannose (Fig. 3B, S2B). Furthermore, upregulation of the UPR transcription factor C/EBP-homologous protein 10 (CHOP) by 2-DG was reduced in combination with Moxe, but remained elevated compared to untreated cells (Fig. 3C and S2C). Mannose completely abrogated the induction of CHOP. Despite the arrest of protein synthesis by Moxe, which likely reduces the amount of unfolded proteins in the ER, selective UPR-induced targets were still expressed following the combination of 2-DG and Moxe, suggesting active UPR.

To determine whether the synergistic effect was achieved via mitochondrial apoptosis, we measured the mitochondrial membrane potential using JC-1. An increase of the ratio of green to red fluorescence indicates depolarization and thus mitochondrial damage (Fig. 3D, E). 2-DG significantly enhanced Moxe-induced mitochondrial depolarization in a concentration-dependent manner. Effects of 2-DG exceeded calculated additivity, suggesting synergistic mitochondrial outer membrane permeabilization (MOMP). This was confirmed by analysis of cleavage of poly-ADP-ribosyltransferase (PARP) in Jeko-1 and Ramos (Fig. 3F and S3A). The combination induced a significantly stronger PARP cleavage than either drug alone, which also exceeded the calculated additive effects. Addition of mannose reduced cleavage of PARP. 2-DG and Moxe each are known to induce intrinsic apoptosis by reduction of MCL-1 [31, 33]. In line, Moxe or 2-DG alone reduced MCL-1 levels in JeKo-1 and Ramos (Fig. 3G and S3B). In combination, reduction of MCL-1 was enhanced, which was calculated as additive effect. Addition of mannose only partially reversed MCL-1 downregulation by 2-DG and by the combination. Thus, decrease of MCL-1 alone does likely not explain synergy. The combination of 2-DG and Moxe had no effects on levels of BCL-2 or of BCL-XL over either drug alone (Fig. S3C, D). Because proteins involved in intrinsic apoptosis did not correlate with synergy, we next analyzed BH3-interacting domain death agonist (BID), which links extrinsic and intrinsic apoptosis [34, 35]. 2-DG induced a reduction in full-length BID, which was synergistically enhanced in combination with Moxe (Fig. 3H and S3E). Addition of mannose completely reversed effects on BID by 2-DG and by the combination. In line with previous reports [36, 37], cleaved tBID was not detectable. However, small and undetectable amounts of short-lived tBID are sufficient to induce cytochrome C release [38].

In summary, synergistic cell death after Moxe and 2-DG correlates with persisting UPR and mitochondrial depolarization, which may be the result of additive reduction of MCL-1, together with synergistic reduction of BID.

### Synergy depends on the IRE1α-XBP1s axis

**Fig. 4:**
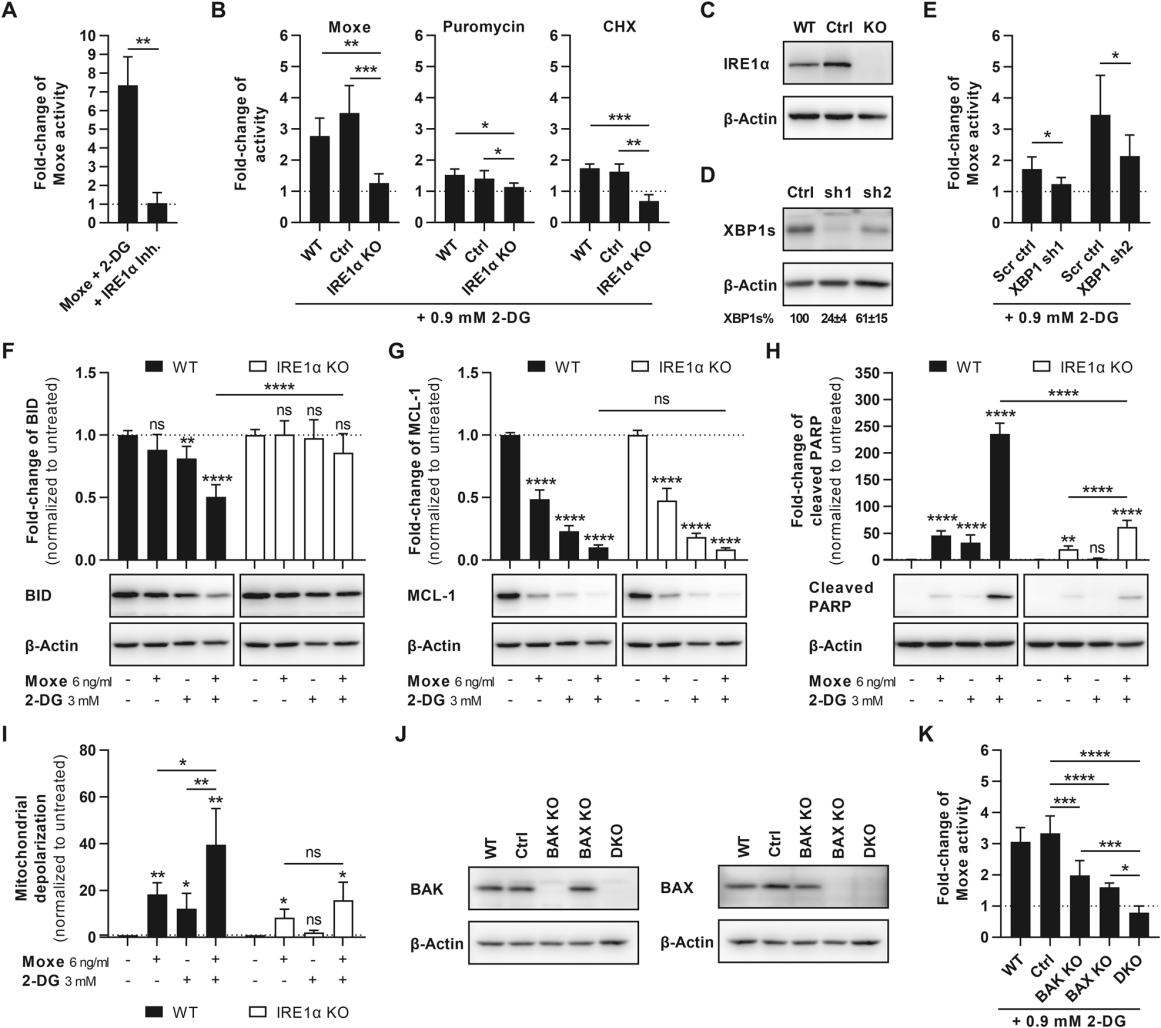
IRE1α is needed for synergistic mitochondrial cell death. **A** JeKo-1 was pre-treated with IRE1α inhibitors (inh.) KIRA6 (1 µM) and STF-083010 (33 µM) for 72 h or remained untreated, before cells were treated with Moxe and 0.9 mM 2-DG. After another 72 h, viability was analyzed by flow cytometry. Shown is the fold-change of Moxe activity (inverse IC_50_), normalized to Moxe alone, as mean + SD of *n* = 3 replicates, *p*-value by unpaired *t*-test. **B** JeKo-1 wild-type (WT) cells and cells carrying a CRISPR control (ctrl) or CRISPR/Cas9-mediated knock-out (KO) of IRE1α were treated with Moxe (*n* = 4), puromycin (*n* = 4), or cycloheximide (CHX, *n* = 3) and 2-DG. Shown are fold-changes of activity, normalized to the respective drug alone, as mean + SD, *p*-values by paired *t*-test. **C** IRE1α KO in JeKo-1 was confirmed by western blot compared to WT and control cells. **D** ShRNA-mediated knock-down of XBP1 in JeKo-1 treated with 2-DG (3 mM) was measured by western blot and quantified by densitometry. Shown are relative expression levels compared to scrambled shRNA control (scr ctrl) as mean ± SD of *n* = 3 replicates. **E** Cytotoxicity of Moxe and 2-DG after XBP1 knock-down was analyzed by flow cytometry. Shown is the fold-change of activity normalized to Moxe alone. Each bar represents mean + SD of *n* = 4 replicates including *p*-values by paired *t*-tests. **F**–**I** JeKo-1 WT (black) and IRE1α KO cells (white) were treated with Moxe and 2-DG for 16 h. **F**–**H** Protein levels of BID (**F**; *n* = 6), MCL-1 (**G**; *n* = 4), and cleaved PARP (**H**; *n* = 6) by western blot were quantified by densitometry. Bars represent mean + SD. *P*-values were determined by ordinary one-way ANOVA (Šídák’s test). **I** Mitochondrial depolarization was determined by flow cytometry as ratio of green to red emitting JC-1. Bars represent mean + SD of *n* = 6 replicates, *p*-values by RM one-way ANOVA (Šídák’s test). **J** CRISPR/Cas9-mediated single or double KO (DKO) of BAK and BAX in JeKo-1 was confirmed by western blot compared to WT and control cells. **K** JeKo-1 WT, ctrl, and BAK/BAX KO cells were treated with Moxe and 2-DG. Shown are fold-changes of activity, normalized to Moxe alone, as mean + SD of *n* = 5 replicates, *p*-values by ordinary one-way ANOVA (Šídák’s test). Fold-change of activity >1 indicates synergy according to Bliss independence (**A**, **B**, **E**, **K**). *P*-values: not significant (ns): *p* > 0.05, *p ≤ 0.05, **p ≤ 0.01, ***p ≤ 0.001, ****p ≤ 0.0001.

To identify the signaling pathway responsible for drug synergy, we knocked-down the proteins IRE1α, PERK, ATF4, and CHOP, which are central to UPR (Fig. S4A, B). However, only IRE1α-targeting shRNAs reversed the synergy of Moxe and 2-DG in JeKo-1 (Fig. S4C). In line with the IRE1α knock-down, simultaneous pharmacological inhibition of both RNase and kinase activity of IRE1α resulted in abolishment of the synergy of Moxe and 2-DG (Fig. 4A). Therefore, we performed a CRISPR/Cas9-mediated knock-out (KO) of IRE1α in JeKo-1 to confirm IRE1α as key driver of synergy (Fig. 4B, C). Compared to control cells, IRE1α KO abolished the synergy of 2-DG and the protein synthesis inhibitors Moxe, puromycin, and CHX. To discriminate impact on synergy, we first knocked-down the RNase-target XBP, which reduced synergy compared to control cells (Fig. 4D, E). Assessing involvement of the IRE1α kinase, we did not find increased phosphorylation of the downstream targets c-Jun N-terminal kinase (JNK), p38 mitogen-activated protein kinase (p38-MAPK), or BCL-2 after the combination compared to either agent alone (Fig. S4D) [39]. Furthermore, pharmacological inhibition of apoptosis signal-regulating kinase 1 (ASK1) did not reduce synergy of Moxe and 2-DG (Fig. S4E) [40]. Taken together, these results suggest that the IRE1α-XBP1s axis acts as driver of synergistic cell death after the combination of 2-DG and inhibitors of protein synthesis.

Although protein synthesis can still be blocked by Moxe in IRE1α KO cells, the KO of IRE1α blocked the reduction of BID after 2-DG and the combination, indicating that IRE1α is actively involved in decreasing total BID (Fig. 4F). In contrast, MCL-1 levels still additively declined in KO cells similar to WT cells after Moxe and 2-DG (Fig. 4G). In accordance with reversed effects on BID, cleavage of PARP was significantly reduced in KO cells compared to WT cells after combination of Moxe and 2-DG, but was still increased compared to Moxe alone (Fig. 4H). Hence, the remaining effects of 2-DG on cell viability in IRE1α KO cells may be explained via additive reduction of MCL-1. In addition to mechanistic data by western blot, KO of IRE1α prevented 2-DG-induced mitochondrial depolarization and significant synergy with Moxe, supporting mitochondrial apoptosis as cause of synergistic cell death (Fig. 4I). However, to prove destabilization of the mitochondrial membrane as crucial event in synergistic cell death, single and double KOs of the pore-forming proteins BAK and BAX were performed (Fig. 4J). Single KO of BAK or BAX significantly reduced synergy compared to control cells, while synergy was abrogated in cells lacking both BAK and BAX, establishing that the drug synergy critically depends on MOMP (Fig. 4K).

### 2-DG enhances immunotoxins in a variety of cancer entities

**Fig. 5:**
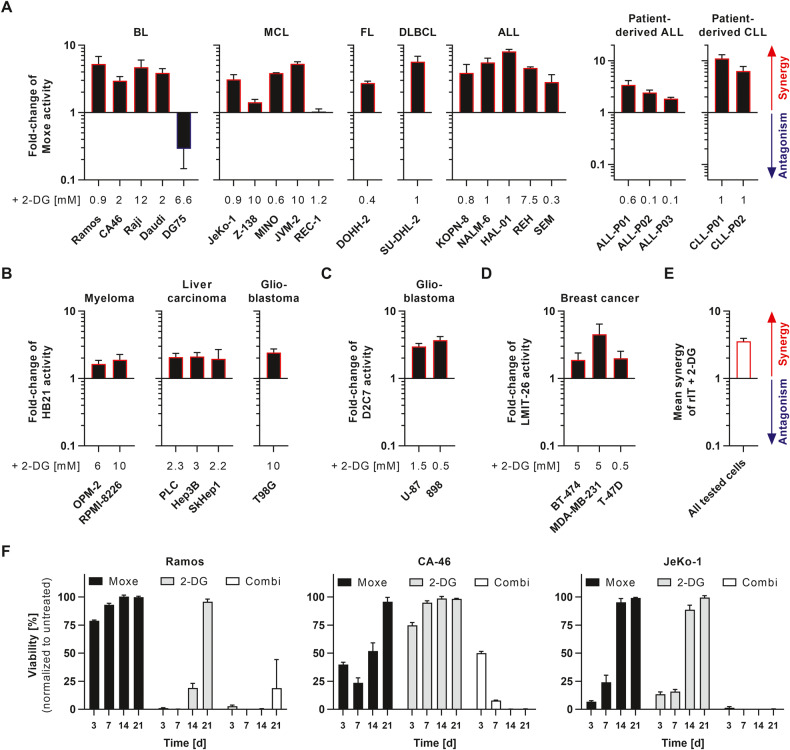
Synergy is broadly reproducible and impairs tumor regrowth over time. **A**–**D** Cell lines (*n* ≥ 3 replicates) and primary cells (*n* ≥ 2) were treated with recombinant immunotoxin (rIT) and indicated concentrations of 2-DG, cell viability measured by flow cytometry (**A**, **B**, **D**) or by WST-8 assay (**C**), and activity (inverse IC_50_) was normalized to rIT alone. Bars represent means + SEM. Activity of (**A**) Moxe against Burkitt’s lymphoma (BL), mantle cell lymphoma (MCL), follicular lymphoma (FL), diffuse-large B cell lymphoma (DLBCL), acute lymphoblastic leukemia (ALL), patient-derived ALL, and patient-derived chronic lymphocytic leukemia (CLL) is shown. Activity of (**B**) transferrin-targeting HB21 against multiple myeloma, liver carcinoma, and glioblastoma, of (**C**) EGFR-targeting D2C7 against glioblastoma, and of (**D**) HER-2-targeting LMIT-26 against breast cancer are shown. **E** summarizes overall synergy + SEM. Fold-change of activity >1 indicates synergy and <1 indicates antagonism according to Bliss independence (**A**–**E**). **F** Ramos, CA-46, and JeKo-1 were treated with Moxe (50/10/20 ng/ml) and/or 2-DG (8/20/6 mM). After 72 h, cells were washed and replated. Viability was analyzed at indicated time points by flow cytometry and normalized to untreated control. Bars represent mean + SD of *n* = 3 replicates.

Immunotoxins are the only approved target cell-specific inhibitors of protein synthesis, and thus offer a potential therapeutic window. We screened immunotoxins in combination with 2-DG against various cell lines and primary patient-derived cells (Fig. 5). The combination of CD22-targeted Moxe and 2-DG showed synergy against 15 out of 17 B cell malignancy cell lines including BL, MCL, follicular lymphoma (FL), diffuse large B cell lymphoma (DLBCL), B cell acute lymphoblastic leukemia (ALL), and five patient-derived ALL and chronic lymphocytic leukemia (CLL) cells (Fig. 5A). The combination had antagonistic effects against DG75 and was additive against REC-1 (Fig. 5A and S5). Strong antagonism in DG-75 may be overestimated as these cells are quickly fragmented once they die after combination treatment, and thus are lost during washing steps, possibly leading to a falsely high percentage of viable cells. To test synergy in malignancies not expressing CD22, we used the human transferrin-targeted immunotoxin HB21. 2-DG synergistically augmented HB21 against two multiple myeloma cell lines, against three liver carcinoma cell lines, and against one glioblastoma cell line (Fig. 5B). The immunotoxin D2C7, which targets both EGFRwt and EGFRvIII, induced synergy in the glioblastoma cell lines U-87 expressing EGFRwt and its variant 898 expressing EGFRvIII (Fig. 5C). Three different HER-2-positive breast cancer cell lines were treated with the HER-2-targeting immunotoxin LMIT-26, which was synergistically enhanced by 2-DG (Fig. 5D). Overall average synergy was 3.6-fold (Fig. 5E). Although Fig. 5A–E only show changes in IC_50_, the combination not only induced a relative curve shift, meaning already sensitive cells got even more sensitive to each drug, but also substantially increased the absolute cell killing at higher concentrations, suggesting partially resistant cells were sensitized to the arrest of protein synthesis (Figs. S5 and S6). These results suggest broad activity of the combination of immunotoxins and 2-DG across various hematologic and solid malignancies.

Cell lines grown in culture often contain a fraction of cells that represents a stem-like population harboring drug resistance even at high concentrations. These cells survive drug exposure and eventually grow out quickly after the treatment. To test whether the combination was superior to either agent alone in attacking the stem-like population, we treated the cell lines Ramos, CA-46, and JeKo-1 with Moxe and 2-DG at concentrations ten times higher than the IC_50_ for 3 days, and then followed viability over time (Fig. 5F). Cells treated with either drug alone regrew quickly, whereas the combination led to a sustained response. While Ramos started to regrow at day 21, CA-46 and JeKo-1 did not reappear within the observational period. Hence, the combination of Moxe and 2-DG not only substantially enhances cell death, but may also sensitize a cell fraction that is more resistant to either drug alone.

### UPR inducers enhance in vivo efficacy

**Fig. 6:**
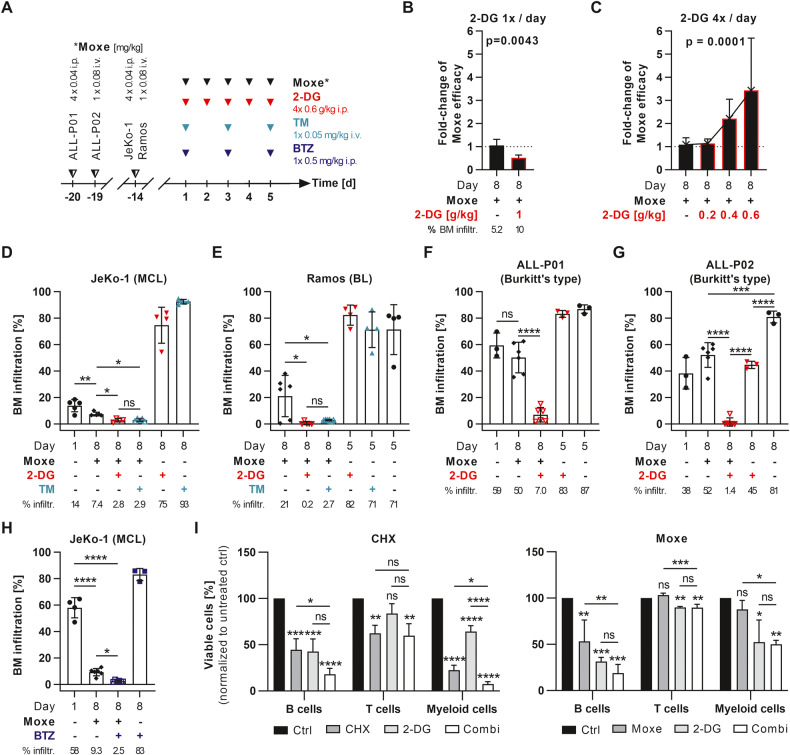
Targeted arrest of protein synthesis generates a therapeutic window in vivo. **A** shows in vivo treatment schedules. JeKo-1, Ramos, or patient-derived ALL were intravenously (i.v.) injected at indicated days. Optimized doses of Moxe were given intraperitoneally (i.p.) or i.v., 2-DG was administered i.p. as single dose (**B**), 4 x per day as indicated (**C**) or 4 × 0.6 g/kg/d (**D**–**G**), tunicamycin (TM) was injected i.v., and bortezomib (BTZ) i.p. Mice were euthanized at indicated days and bone marrow (BM) infiltration rate was determined by flow cytometry. **B**, **C** BM infiltration normalized to Moxe alone (=Moxe efficacy) is shown as mean + SD of *n* = 4–10 mice. *P*-values were calculated by unpaired *t*-test (**B**) and linear trend by ordinary one-way ANOVA (**C**). **D**–**G** show mean BM infiltration ± SD of *n* = 3–6 mice with *p*-values by ordinary one-way ANOVA (Šídák’s test). **H** JeKo-1 bearing mice were treated with Moxe and BTZ. Each bar represents mean BM infiltration ± SD of *n* = 4–5 mice including *p*-values by ordinary one-way ANOVA (Šídák’s test). **I** PBMCs isolated from healthy donors were treated with 1 µg/ml cycloheximide (CHX, B and T cells), 0.4 µg/ml CHX (myeloid cells), 100 ng/ml Moxe, and 3 mM 2-DG. Rate of viable CD19^+^ B cells, CD3^+^ T cells, or CD11b^+^ myeloid cells was analyzed by flow cytometry and normalized to untreated control (ctrl). Bars show mean + SD of *n* = 3 replicates. *P*-values were determined by ordinary one-way ANOVA (Šídák’s test). *P*-values: not significant (ns): *p* > 0.05, *p ≤ 0.05, **p ≤ 0.01, ***p ≤ 0.001, ****p ≤ 0.0001.

Finally, we investigated whether combination of Moxe and 2-DG can be exploited for in vivo treatment of B cell malignancies. To facilitate the study of synergy, Moxe doses were optimized so that the immunotoxin alone achieved not more than a six-fold reduction of tumor infiltration. Therefore, Moxe was injected up to four times per day for 5 consecutive days to maintain immunotoxin blood levels over time (Fig. 6A) [41]. Because 2-DG has been injected exclusively as daily bolus doses in mice, we initially treated 2-DG accordingly [42–44]. In a systemic JeKo-1 mouse model, daily boli of 1 g/kg 2-DG did not enhance but reduce Moxe efficacy (Fig. 6B). Because 2-DG has a very short half-life in vivo, and in vitro data suggested a 2-DG exposure over 24 h was needed to induce synergy, we injected 2-DG four times daily for 5 days (high-frequent (HF) 2-DG), which induced a dose-dependent increase of Moxe efficacy (Fig. 6C). This also increased the maximally tolerated dose (MTD) of 2-DG over 5 days from a total of 5 g/kg for daily single doses (1 g/kg/dose) to 12 g/kg for HF-2-DG (0.6 g/kg/dose). We then compared enhancement of Moxe by optimized HF-2-DG and by TM. Against JeKo-1, 2-DG and TM similarly enhanced Moxe-induced reduction of bone marrow (BM) infiltration by 3-fold, although neither 2-DG nor TM alone had any effects on BM infiltration (Fig. 6D). In a systemic Ramos model, 2-DG enhanced Moxe by 35-fold and TM by 8-fold (Fig. 6E). The combination was also highly effective against two patient-derived ALL xenografts showing a 7- and 37-fold enhancement of Moxe by 2-DG (Fig. 6F, G). To test for potential clinical applicability, we found that BTZ was able to enhance Moxe efficacy by 4-fold against JeKo-1 in vivo (Fig. 6H) [45].

Lastly, peripheral blood mononuclear cells (PBMCs) of healthy human donors were treated with CHX or Moxe with or without 2-DG and cell subsets were analyzed by flow cytometry (Fig. 6I). Compared to untreated control, CHX unspecifically reduced viable B cells, T cells, and myeloid cells. 2-DG alone reduced viable B cells and myeloid cells, but did not affect T cells. In combination, synergy was only detected against myeloid cells. In contrast, CD22-targeted immunotoxin Moxe did not reduce viable T cells or myeloid cells and thus, cytotoxicity of 2-DG against T cells or myeloid cells was not enhanced by Moxe. As for CHX, effects of the combination on B cells were only additive.

Taken together, enhancement of UPR-induced cell death by a targeted arrest of protein synthesis using immunotoxins is highly synergistic also in vivo and provides a therapeutic window via two independent means, which strongly supports clinical evaluation of the combination.

## Discussion

The aim of our study was to block the upregulation of proteins which rescue a cell from apoptosis after a stress response using cell-selective inhibitors of protein synthesis. We find highly synergistic cytotoxicity in a large number of tumor cell lines, primary patient-derived ALL and CLL cells, and mouse models.

### IRE1α is essential for synergistic cell death

**Fig. 7:**
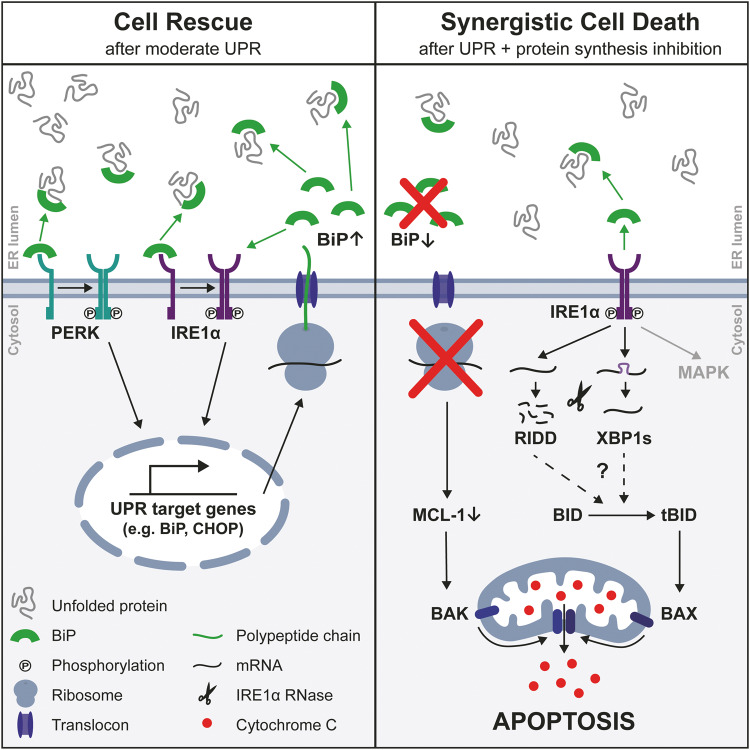
Proposed mechanism how the block of UPR counter-regulation by protein synthesis inhibition induces synergistic mitochondrial apoptosis. BiP dissociates from UPR receptors IRE1α and PERK to bind accumulating unfolded proteins in the ER lumen leading to active UPR and expression of target genes like BiP and CHOP. Upregulated BiP binds UPR receptors to turn off their signal, which resolves UPR and prevents cell death. Additional block of protein synthesis by itself induces reduction of MCL-1 and blocks upregulation of BiP. Maintained UPR induces IRE1α-dependent cleavage of BID, which, together with reduced MCL-1, induces BAX/BAK pore formation and mitochondrial apoptosis. UPR: unfolded protein response, ER: endoplasmic reticulum, RIDD: regulated IRE1α-dependent decay.

To analyze the underlying mechanism, we used the reversible UPR inducer 2-DG in combination with immunotoxins, which are the only clinically approved inhibitors of protein synthesis (Fig. 7). Accumulation of unfolded proteins in the ER lumen recruits BiP away from the UPR signaling receptors and activates UPR [4–7]. Cell death after UPR is counteracted by upregulation of BiP, which terminates the distress signal by reoccupying UPR receptors [4, 5, 15–17]. Preventing BiP upregulation by an additional block of protein synthesis leads to maintained UPR signaling and to execution of UPR-induced apoptosis. That arrest of protein synthesis by several drugs synergistically enhances 2-DG-induced cell death suggests absence of a protective protein such as upregulated BiP rather than UPR-induced upregulation of an executor of cell death as cause of the synergy. The current literature agrees on mitochondrial apoptosis as the common form of cell death after UPR [4, 9, 18, 34]. Reported upstream molecules responsible for mitochondrial apoptosis differ depending on cell type and UPR-causing drug [4, 18]. Here, IRE1α was identified as the key mediator of 2-DG-induced cell death and the synergy with Moxe, CHX, and puromycin as effects are reduced by knock-down and are abrogated by knock-out of IRE1α. Exploring BCL-2 family members to identify the executors of synergistic cell death, we confirmed reduction of anti-apoptotic MCL-1 after 2-DG and Moxe [31, 33]. MCL-1 is rapidly degraded due to its PEST-sequence [30] after protein synthesis is arrested by Moxe via ADP-ribosylation of eEF2 and after 2-DG via UPR-activated PERK, which phosphorylates eukaryotic initiation factor 2α (eIF2α) [29, 33]. In line with the similar causal mechanism, MCL-1 levels fall additively after combination treatment and independently from IRE1α, indicating that MCL-1 is not responsible for the synergy. In contrast, BID was reduced synergistically after the combination treatment in an IRE1α-dependent way. BID is considered a connection between extrinsic and intrinsic apoptosis and is critical for apoptosis if UPR is not resolved in time [34, 35, 37]. Because tBID activates BAX, it could well explain the synergistic destabilization of mitochondria on top of the reduced MCL-1, which activates BAK, leading to BAX/BAK pore formation, release of mitochondrial cytochrome C, and apoptosis [34, 46, 47]. In line, individual knock-out of BAK and BAX reduced synergistic effects of Moxe and 2-DG, while only the double knock-out completely abolished synergy. BID is likely not reduced by IRE1α’s kinase domain but rather by its RNase domain, which can induce a decay of pro-survival mRNAs and miRNAs via RIDD [10, 48], activating caspase-2-mediated cleavage of BID [37, 49]. Alternatively, caspase 8-mediated cleavage of BID can be induced by upregulation of death receptors or by an incompletely understood IRE1α-dependent pathway [50–52].

### Resistance to UPR-induced cell death—a clinical problem

BTZ is a UPR-inducing agent used to treat MM and MCL. However, one third of MM patients and more than half of MCL patients are resistant to BTZ [53, 54]. Resistance has been linked to an upregulation of heat shock proteins like BiP, HSP27, and HSPB8 [21–23], which regulate ER homeostasis and promote cell survival after UPR. BTZ resistant MCL cells show increased levels of BiP and its co-chaperone HSP90 and inhibition of HSP90 restores sensitivity to BTZ in vitro and in vivo [20]. In line, BiP levels of 2-DG resistant vs. sensitive breast cancer cells are elevated [27] and BiP knock-down sensitizes ALL cells to 2-DG-induced cell death [55]. Apart from chaperones, proteins including UFD1, necessary for degradation of misfolded proteins, protect ALL from UPR-induced apoptosis [56]. These data together with ineffectiveness of UPR inducers as monotherapy support that upregulated protective proteins counteract cytotoxicity of UPR, while a combination with inhibitors of protein synthesis may reverse preexisting resistance to UPR. This concept of preventing a stressor-induced counter-regulation by inhibitors of protein synthesis may also be applicable to oxidative stress or to DNA damage response.

2-DG preclinically shows synergy with chemotherapy and radiation [57–59]. That 2-DG-induced cell death is also reversed by mannose indicates causal relation to UPR similar to our findings [27, 57]. However, clinical studies were designed based on 2-DG as irreversible glycolytic inhibitor rather than UPR inducer, which possibly affected the dose regimen in these studies [59–61]. In line, 2-DG was given orally once per day in combination with docetaxel in advanced solid tumors [62] and with radiotherapy in patients with glioblastoma multiforme [63]. 2-DG alone or in combination had limited clinical efficacy [59, 62, 63]. Taken into consideration that 2-DG has a short half-life in vivo, we propose that a repeated administration as shown in our model may be needed to translate the strong preclinical data with 2-DG in combination treatment towards a higher benefit for patients.

### Clinical relevance of the combination treatment

Inhibition of protein synthesis by immunotoxins in combination with induction of UPR may generate a therapeutic window in two ways. First, we find that PBMCs of healthy donors are sensitive to either drug alone but not to the synergistic effects of the combination. Stronger effects in malignant cells than in healthy cells may be due to high baseline UPR in tumor cells, which have a particularly high demand for proteins [64]. Since protein folding is error-prone [65], tumor cells continuously have to cope with UPR, which offers a unique vulnerability [64, 66]. Secondly, this vulnerability can be exploited by arresting protein synthesis with immunotoxins in a targeted manner, which is distinct from the general ribosome inhibitors CHX, puromycin, or omacetaxine [29, 67, 68]. In line, other approved immunotoxins may benefit from the combination, such as approved Tagraxofusp (Elzonris®) [69], Denileukin Diftitox (Ontark®) [70] or immunotoxins in early phase clinical trials including D2C7 or MOC31PE [71, 72].

With the goal of cell survival, a cellular stress response generally aims to counteract damage. Preventing this counter-regulation by simultaneous inhibition of protein synthesis powerfully induces synergistic cell death in malignant but not in healthy cells. The broad applicability throughout several cancer entities suggests a tumor-cell intrinsic dependency and, together with a cell-targeted inhibition of protein synthesis by immunotoxins, may warrant clinical evaluation of this previously unexplored treatment strategy.

## Materials and methods

### Cell lines

Cells were cultured under standard conditions at 37 °C and 5% CO_2_. All culture media were supplemented with 50 IU/ml penicillin and 50 µg/ml streptomycin (P/S; Gibco, Thermo Fisher Scientific, Waltham, MA, USA). Cell lines of B cell origin were cultured in RPMI 1640 (Gibco), 10% fetal calf serum (FCS, Sigma-Aldrich, Merck, Darmstadt, Germany) and 2 mM L-glutamine (Gibco). Liver carcinoma, glioblastoma and HEK293T were cultured in DMEM (Gibco), 10% FCS, MDA-MB-231 in Leibovitz’s L-15 (Gibco), 10% FCS, and T-47D and BT-474 were cultured in RPMI 1640, 10% FCS, 2 mM L-glutamine, and 10 µg/ml human insulin (Gibco). The stromal cell line OP9 and patient-derived ALL cells were cultured in α-MEM (Gibco), 20% FCS, the murine embryonic liver cells EL08-1D2 in α-MEM (Gibco), 10% FCS (CCPro, Oberdorla, Germany), 10% horse serum (StemCell technologies, Vancouver, Canada), and 10 μM 2-mercaptoethanol (Gibco). The cells were grown on 0.1% gelatin coated cell culture plates. Primary CLL cells were cultured in RPMI 1640, 10% FCS, 1 mM sodium pyruvate, 2 mM L-glutamine, 20 mg/ml L-asparagine, 50 µM 2-mercaptoethanol, 10 mM HEPES, and MEM non-essential amino acids (all from Gibco). Table S1 provides information about the source, authentication, and mycoplasma testing of all cell lines.

### Human samples

CLL and ALL samples were collected with informed consent and in accordance with the Helsinki declaration on approval from the Ethics Committee of the University of Erlangen-Nuremberg (Table S2).

PBMCs were isolated from heparinized blood samples by centrifugation over a Ficoll-Hypaque layer (Biochrom, Berlin, Germany) of 1.077 g/ml density. For CLL samples, T cells and monocytes were removed with anti-CD2 and anti-CD14 magnetic beads (Dynabeads M450, Dynal, Oslo, Norway).

### Compounds

The immunotoxins Moxe, HB21, LMIT-26, and D2C7 have been produced following standard protocol [73]. Puromycin was purchased from Thermo Fisher Scientific and CHX from Sigma-Aldrich. 2-DG (≥99% purity), TM, and mannose were purchased from Sigma-Aldrich. BTZ, KIRA6, STF-083010, and selonsertib were purchased from MedChemExpress (Monmouth Junction, NJ, USA).

### Cell assays

For cytotoxicity assays, 100,000–200,000 cells/ml were treated with indicated compounds at various concentrations for up to 72 h. To determine cell death by flow cytometry, cells were stained with 7-AAD (Biolegend, San Diego, CA, USA), measured with FACSCantoII (BD, Franklin Lakes, NJ, USA), and analyzed by FlowJo Software (TreeStar, Ashland, OR, USA). Colorimetric analysis of cell viability was performed by staining with WST-8 (Dojindo Laboratories, Kumamoto, Japan) and measurement of absorbance at 450 nm. IC_50_ concentrations were calculated by non-linear regression using GraphPad Prism v9.0.2. (GraphPad Software, San Diego, CA, USA).

For assays using primary cells, 5000 OP9 stromal cells (for ALL) and 1000 EL08-1D2 cells (for CLL) were plated per well in a 96-well plate. After 24 h, 1 × 10^6^ ALL or CLL cells/ml were added in their appropriate medium followed by indicated concentrations of Moxe and 2-DG 4 h later. 72 h after treatment start, survival was measured by flow cytometry using 7-AAD staining (ALL) or PI staining (CLL).

PBMCs from healthy donors were plated at a concentration of 400,000 cells/ml and were treated with Moxe, CHX, or 2-DG as indicated. After 72 h, cells were stained with anti-human-CD19 (clone HIB19; Biolegend), anti-human-CD3 (clone UCHT1; Biolegend), anti-human-CD11b (clone M1/70; Biolegend), and Zombie Aqua (Biolegend) and subset specific viability was analyzed by flow cytometry.

### Western blot and antibodies

For protein analysis, cells were lysed in modified RIPA buffer (50 mM Tris pH 7.4 (Carl Roth, Karlsruhe, Germany), 1% NP40 (Roche, Mannheim, Germany), 0.25% sodium deoxycholate (Sigma-Aldrich), 150 mM NaCl (Carl Roth), 1 mM EDTA (Millipore, Merck) and protease and phosphatase inhibitor cocktail (Thermo Fisher Scientific). Proteins were separated on 12% SDS polyacrylamide gel, transferred to polyvinylidene difluoride (PVDF) membranes (Thermo Fisher Scientific) in a tank blot system (Bio-Rad, Hercules, CA, USA) and stained with antibodies against human proteins. Antibodies used recognized ATF4 (#11815), BAK (#12105), BAX (#89477), BCL-2 (#15071), phospho-BCL-2 (#2827), BCL-XL (#2764), BID (#2002), BiP (#3177), CHOP (#2895), IRE1α (#3294), phospho-JNK (#4668), MCL-1 (#94296), cleaved PARP (#5625), PERK (#5683), phospho-p38 MAPK (#9234), XBP1s (#12782) (all from Cell Signaling Technology, Danvers, MA, USA), and β-Actin (#8224, Abcam, Cambridge, UK). Secondary anti-mouse/rabbit antibodies conjugated to HRP were from Santa Cruz Biotechnology (Dallas, TX, USA). Chemiluminescence was measured on Amersham™ Imager 600 (Cytiva, Marlborough, MA, USA) following application of SignalFire™ ECL Reagent (Cell Signaling Technology), ECL™ Prime Western Blotting System (Cytiva), or SuperSignal™ West Femto Maximum Sensitivity Substrate (Thermo Fisher Scientific). Relative protein amount was quantified with ImageJ and normalized to the respective β-Actin band. Uncropped blots and respective molecular weight markers are shown in the Supplementary Information.

### RT-PCR for XBP1 mRNA splicing

Total RNA was isolated from cells by RNeasy® Mini Kit (Qiagen, Hilden, Germany) and 1 µg RNA was reverse transcribed by using QuantiTect® Reverse Transcription Kit (Qiagen). cDNA was amplified by 5PRIME HotMaster® Taq DNA Polymerase (Quantabio, Beverly, MA, USA) using primers specific for human XBP1 resulting in a 200 bp PCR product for XBP1 and 174 bp product for spliced XBP1s (forward primer: 5’-AGAACCAGGAGTTAAGACAG-3’, reverse primer: 5‘-AATACCGCCAGAATCCATG-3’, Eurofins, Luxemburg, Luxemburg). PCR products were separated on a 12% TBE polyacrylamide gel, stained with Midori Green Advance (Nippon Genetics, Düren, Germany), and visualized on a ChemiDoc XRS+ machine with ImageLab software (Bio-Rad). Ratio of XBP1s to XBP1 was quantified by ImageJ. Uncropped gels and respective molecular weight markers are shown in the Supplementary Information.

### Mitochondrial membrane potential

To measure mitochondrial membrane potential, cells were stained with 2 µM JC-1 (MedChemExpress) for 20 min at 37 °C, washed, and analyzed by flow cytometry using 488 nm excitation.

### Lentiviral transduction

For lentivirus production, HEK293T cells were transfected using FuGENE®6 (Promega, Madison, WI, USA) with psPAX2 (Addgene plasmid #12260), pMD2.G (Addgene plasmid #12259), and the respective transfer plasmid in an equimolar ratio. Medium containing lentiviral particles was harvested and concentrated 20-fold by Lenti-X™ Concentrator (Takarabio, Kusatsu, Japan). JeKo-1 was transduced with a MOI of 3. Polybrene (Sigma-Aldrich) at 8 µg/ml was added and cells centrifuged at 1200 × *g* for 2 h at 32 °C.

### shRNA-mediated knock-down

Of the lentiviral vector pLKO.1 puro (Addgene plasmid #8453 [74]) puromycin resistance was exchanged with eGFP. Following RNAi Consortium (Broad Institute) recommendations, single-stranded oligos were ordered from Eurofins, annealed, and inserted into pLKO.1 eGFP after digestion with AgeI and EcoRI (NEB, Ipswich, MA, USA). After lentiviral transduction of target cells, top 5% of GFP^+^ cells were sorted using MoFlo Astrios EQ (Beckman Coulter, Brea, CA, USA) or MoFlo XDP (Beckman Coulter). The following shRNA’s have been used: scrambled shRNA: SHC007, IRE1α shRNA1: TRCN0000000529, IRE1α shRNA2: TRCN0000000528, XBP1 shRNA1: TRCN0000019806, XBP1 shRNA2: TRCN0000019804, PERK shRNA: TRCN0000262380, CHOP shRNA: TRCN0000364393, ATF4 shRNA: TRCN0000013573. For sequences, see Table S3.

### CRISPR/Cas9

Guide RNA (gRNA) sequences for IRE1α and BAK KO come from the human GeCKO v2 library [75], while gRNA against BAX was designed by CHOPCHOP [76]. gRNA oligos (see Table S3) were ordered from Eurofins, annealed, and inserted in the lentiCRISPRv2GFP vector (Addgene plasmid #82416 [77]) at the BsmBI cloning site. JeKo-1 cells were lentivirally transduced, sorted for GFP^+^ cells, and single cells cloned by limited dilution. Clones were screened for successful KOs by western blot and allelic sequencing.

### Animal studies

Animal studies were approved by the local regulatory agency (government of Unterfranken). Animals were handled according to institutional guidelines. Experiments were conducted in a randomized and non-blinded manner.

Either ten million JeKo-1 cells, five million Ramos cells, or two million patient-derived ALL cells (ALL-P01 or ALL-P02) were injected i.v. in the tail vain of 6 to 8 weeks old NSG mice (NOD.Cg-Prkdc^scid^ Il2rg^tm1Wjl^/SzJ). Treatment start of mice bearing JeKo-1 or Ramos was after 14 days, of ALL-P01 after 20 days, and of ALL-P02 after 19 days. All in vivo administered drugs have been dissolved and diluted in sterile PBS (Gibco). Moxe was injected four times daily for 5 consecutive days at 0.04 mg/kg i.p. for JeKo-1 and ALL-P02 or once per day at 0.08 mg/kg i.v. for Ramos and ALL-P01. 2-DG was given either at 1 g/kg i.p. once per day or at a dose of 0.2, 0.4, or 0.6 g/kg i.p. four times a day for five consecutive days. MTD of 2-DG was defined as the highest applicable dose that did not produce central nervous side effects (changes in behavior). TM was injected i.v. at a dose of 0.05 mg/kg and BTZ i.p. at a dose of 0.5 mg/kg once per day every other day. Three days after the last injection of immunotoxin or when predefined termination criteria were fulfilled (hind limb paralysis), animals were humanely euthanized. BM was extracted by flushing femurs with a syringe or by crushing spine with a mortar and pestle. Single cell suspensions were obtained by mashing through a 40 µm cell strainer. Tumor infiltration of cells derived from bone marrow was analyzed by flow cytometry after Fc blocking with anti-murine CD16/CD32 (Biolegend) and staining with Zombie Aqua (Biolegend) and anti-human CD20 (clone 2H7; for JeKo-1 and Ramos; Biolegend), or anti-human CD19 (clone HIB19) and anti-human CD22 (clone HIB22; for patient-derived ALL; Biolegend).

### Statistics and data analysis

Sample size of in vitro experiments was not determined by a priori statistics. Sample size of in vivo experiments was estimated by a priori Wilcoxon-Mann-Whitney tests using G*Power v3.1.9.4 [78]. Number of animals and number of replicates for patient-derived cells was limited due to availability. Bone marrow samples derived from mice at end stage disease rarely presented with a viability of less than 5% and thus were excluded from analysis (viability for all other samples was >70%). Data were analyzed in GraphPad Prism v9.0.2. (GraphPad Software). Results are shown as mean ± standard deviation (SD) or standard error of the mean (SEM). Statistical significance (*p* < 0.05) of two groups was determined by two-tailed *t*-test and multiple comparisons were performed by one-way ANOVA. The number of independent replicates and the statistical test used for each experiment are indicated in the respective figure legend. Additivity was calculated by Bliss independence as E_A+B_ = E_A_ + E_B_ – E_A_ E_B_ [32].

## Supplementary information

Supplementary Figure Legends

Supplementary Figure S1

Supplementary Figure S2

Supplementary Figure S3

Supplementary Figure S4

Supplementary Figure S5

Supplementary Figure S6

Supplementary Table S1

Supplementary Table S2

Supplementary Table S3

Original Western Blots and DNA Gels

Reproducibility Checklist

## Data Availability

All data generated or analyzed during this study are included in this published article and its supplementary information files.

## References

[1] Galluzzi L, Yamazaki T, Kroemer G (2018). Linking cellular stress responses to systemic homeostasis. Nat Rev Mol Cell Biol.

[2] Galluzzi L, Bravo-San Pedro JM, Kepp O, Kroemer G (2016). Regulated cell death and adaptive stress responses. Cell Mol Life Sci.

[3] Ron D, Walter P (2007). Signal integration in the endoplasmic reticulum unfolded protein response. Nat Rev Mol Cell Biol.

[4] Hetz C, Zhang K, Kaufman RJ (2020). Mechanisms, regulation and functions of the unfolded protein response. Nat Rev Mol Cell Biol.

[5] Bertolotti A, Zhang Y, Hendershot LM, Harding HP, Ron D (2000). Dynamic interaction of BiP and ER stress transducers in the unfolded-protein response. Nat Cell Biol.

[6] Oikawa D, Kimata Y, Kohno K, Iwawaki T (2009). Activation of mammalian IRE1alpha upon ER stress depends on dissociation of BiP rather than on direct interaction with unfolded proteins. Exp Cell Res.

[7] Walter P, Ron D (2011). The unfolded protein response: from stress pathway to homeostatic regulation. Science..

[8] Shen J, Chen X, Hendershot L, Prywes R (2002). ER stress regulation of ATF6 localization by dissociation of BiP/GRP78 binding and unmasking of Golgi localization signals. Dev Cell.

[9] Hetz C (2012). The unfolded protein response: controlling cell fate decisions under ER stress and beyond. Nat Rev Mol Cell Biol.

[10] Hollien J, Weissman JS (2006). Decay of endoplasmic reticulum-localized mRNAs during the unfolded protein response. Science..

[11] Harding HP, Zhang Y, Ron D (1999). Protein translation and folding are coupled by an endoplasmic-reticulum-resident kinase. Nature..

[12] Haze K, Yoshida H, Yanagi H, Yura T, Mori K (1999). Mammalian transcription factor ATF6 is synthesized as a transmembrane protein and activated by proteolysis in response to endoplasmic reticulum stress. Mol Biol Cell.

[13] Yoshida H, Matsui T, Yamamoto A, Okada T, Mori K (2001). XBP1 mRNA is induced by ATF6 and spliced by IRE1 in response to ER stress to produce a highly active transcription factor. Cell..

[14] Vattem KM, Wek RC (2004). Reinitiation involving upstream ORFs regulates ATF4 mRNA translation in mammalian cells. Proc Natl Acad Sci USA.

[15] Amin-Wetzel N, Neidhardt L, Yan Y, Mayer MP, Ron D (2019). Unstructured regions in IRE1alpha specify BiP-mediated destabilisation of the luminal domain dimer and repression of the UPR. Elife..

[16] Amin-Wetzel N, Saunders RA, Kamphuis MJ, Rato C, Preissler S, Harding HP (2017). A J-protein Co-chaperone recruits BiP to monomerize IRE1 and repress the unfolded protein response. Cell..

[17] Pincus D, Chevalier MW, Aragon T, van Anken E, Vidal SE, El-Samad H (2010). BiP binding to the ER-stress sensor Ire1 tunes the homeostatic behavior of the unfolded protein response. PLoS Biol.

[18] Iurlaro R, Munoz-Pinedo C (2016). Cell death induced by endoplasmic reticulum stress. FEBS J.

[19] Pihan P, Carreras-Sureda A, Hetz C (2017). BCL-2 family: integrating stress responses at the ER to control cell demise. Cell Death Differ.

[20] Roue G, Perez-Galan P, Mozos A, Lopez-Guerra M, Xargay-Torrent S, Rosich L (2011). The Hsp90 inhibitor IPI-504 overcomes bortezomib resistance in mantle cell lymphoma in vitro and in vivo by down-regulation of the prosurvival ER chaperone BiP/Grp78. Blood..

[21] Mozos A, Roué G, López-Guillermo A, Jares P, Campo E, Colomer D (2011). The expression of the endoplasmic reticulum stress sensor BiP/GRP78 predicts response to chemotherapy and determines the efficacy of proteasome inhibitors in diffuse large b-cell lymphoma. Am J Pathol.

[22] Chauhan D, Li G, Shringarpure R, Podar K, Ohtake Y, Hideshima T (2003). Blockade of Hsp27 overcomes Bortezomib/proteasome inhibitor PS-341 resistance in lymphoma cells. Cancer Res.

[23] Hamouda MA, Belhacene N, Puissant A, Colosetti P, Robert G, Jacquel A (2014). The small heat shock protein B8 (HSPB8) confers resistance to bortezomib by promoting autophagic removal of misfolded proteins in multiple myeloma cells. Oncotarget.

[24] Heifetz A, Keenan RW, Elbein AD (1979). Mechanism of action of tunicamycin on the UDP-GlcNAc:dolichyl-phosphate Glc-NAc-1-phosphate transferase. Biochemistry.

[25] Kurtoglu M, Maher JC, Lampidis TJ (2007). Differential toxic mechanisms of 2-deoxy-D-glucose versus 2-fluorodeoxy-D -glucose in hypoxic and normoxic tumor cells. Antioxid Redox Signal.

[26] Obeng EA, Carlson LM, Gutman DM, Harrington WJ, Lee KP, Boise LH (2006). Proteasome inhibitors induce a terminal unfolded protein response in multiple myeloma cells. Blood.

[27] Kurtoglu M, Gao N, Shang J, Maher JC, Lehrman MA, Wangpaichitr M (2007). Under normoxia, 2-deoxy-D-glucose elicits cell death in select tumor types not by inhibition of glycolysis but by interfering with N-linked glycosylation. Mol Cancer Ther.

[28] Dmitriev SE, Vladimirov DO, Lashkevich KA (2020). A quick guide to small-molecule inhibitors of eukaryotic protein synthesis. Biochemistry (Moscow).

[29] Pastan I, Hassan R, Fitzgerald DJ, Kreitman RJ (2006). Immunotoxin therapy of cancer. Nat Rev Cancer.

[30] Kozopas KM, Yang T, Buchan HL, Zhou P, Craig RW (1993). MCL1, a gene expressed in programmed myeloid cell differentiation, has sequence similarity to BCL2. Proc Natl Acad Sci USA.

[31] Du X, Youle RJ, FitzGerald DJ, Pastan I (2010). Pseudomonas exotoxin A-mediated apoptosis is Bak dependent and preceded by the degradation of Mcl-1. Mol Cell Biol.

[32] Bliss CI (1939). The toxicity of poisons applied jointly. Ann Appl Biol.

[33] Tailler M, Lindqvist LM, Gibson L, Adams JM (2019). By reducing global mRNA translation in several ways, 2-deoxyglucose lowers MCL-1 protein and sensitizes hemopoietic tumor cells to BH3 mimetic ABT737. Cell Death Differ.

[34] Tait SW, Green DR (2010). Mitochondria and cell death: outer membrane permeabilization and beyond. Nat Rev Mol Cell Biol.

[35] Kantari C, Walczak H (2011). Caspase-8 and bid: caught in the act between death receptors and mitochondria. Biochim Biophys Acta.

[36] Bonzon C, Bouchier-Hayes L, Pagliari LJ, Green DR, Newmeyer DD (2006). Caspase-2-induced apoptosis requires bid cleavage: a physiological role for bid in heat shock-induced death. Mol Biol Cell.

[37] Upton JP, Austgen K, Nishino M, Coakley KM, Hagen A, Han D (2008). Caspase-2 cleavage of BID is a critical apoptotic signal downstream of endoplasmic reticulum stress. Mol Cell Biol.

[38] Zha J, Weiler S, Oh KJ, Wei MC, Korsmeyer SJ (2000). Posttranslational N-myristoylation of BID as a molecular switch for targeting mitochondria and apoptosis. Science.

[39] Urano F, Wang X, Bertolotti A, Zhang Y, Chung P, Harding HP (2000). Coupling of stress in the ER to activation of JNK protein kinases by transmembrane protein kinase IRE1. Science.

[40] Nishitoh H, Matsuzawa A, Tobiume K, Saegusa K, Takeda K, Inoue K (2002). ASK1 is essential for endoplasmic reticulum stress-induced neuronal cell death triggered by expanded polyglutamine repeats. Genes Dev.

[41] Muller F, Cunningham T, Liu XF, Wayne AS, Pastan I (2016). Wide variability in the time required for immunotoxins to kill B lineage acute lymphoblastic leukemia cells: implications for trial design. Clin Cancer Res.

[42] Maschek G, Savaraj N, Priebe W, Braunschweiger P, Hamilton K, Tidmarsh GF (2004). 2-deoxy-D-glucose increases the efficacy of adriamycin and paclitaxel in human osteosarcoma and non-small cell lung cancers in vivo. Cancer Res.

[43] Beneteau M, Zunino B, Jacquin MA, Meynet O, Chiche J, Pradelli LA (2012). Combination of glycolysis inhibition with chemotherapy results in an antitumor immune response. Proc Natl Acad Sci USA.

[44] Larrue C, Saland E, Vergez F, Serhan N, Delabesse E, Mansat-De Mas V (2015). Antileukemic activity of 2-Deoxy-d-Glucose through Inhibition of N-linked glycosylation in acute myeloid leukemia with FLT3-ITD or c-KIT mutations. Mol Cancer Ther.

[45] Robak T, Huang H, Jin J, Zhu J, Liu T, Samoilova O (2015). Bortezomib-based therapy for newly diagnosed mantle-cell lymphoma. N Engl J Med.

[46] Eskes R, Desagher S, Antonsson B, Martinou JC (2000). Bid induces the oligomerization and insertion of Bax into the outer mitochondrial membrane. Mol Cell Biol.

[47] Willis SN, Chen L, Dewson G, Wei A, Naik E, Fletcher JI (2005). Proapoptotic Bak is sequestered by Mcl-1 and Bcl-xL, but not Bcl-2, until displaced by BH3-only proteins. Genes Dev.

[48] Han D, Lerner AG, Vande Walle L, Upton JP, Xu W, Hagen A (2009). IRE1alpha kinase activation modes control alternate endoribonuclease outputs to determine divergent cell fates. Cell.

[49] Upton JP, Wang L, Han D, Wang ES, Huskey NE, Lim L (2012). IRE1alpha cleaves select microRNAs during ER stress to derepress translation of proapoptotic Caspase-2. Science.

[50] Lam M, Lawrence DA, Ashkenazi A, Walter P (2018). Confirming a critical role for death receptor 5 and caspase-8 in apoptosis induction by endoplasmic reticulum stress. Cell Death Differ.

[51] Munoz-Pinedo C, Lopez-Rivas A (2018). A role for caspase-8 and TRAIL-R2/DR5 in ER-stress-induced apoptosis. Cell Death Differ.

[52] Estornes Y, Aguileta MA, Dubuisson C, De Keyser J, Goossens V, Kersse K (2014). RIPK1 promotes death receptor-independent caspase-8-mediated apoptosis under unresolved ER stress conditions. Cell Death Dis.

[53] Bai Y, Su X (2021). Updates to the drug-resistant mechanism of proteasome inhibitors in multiple myeloma. Asia Pac J Clin Oncol.

[54] Perez-Galan P, Mora-Jensen H, Weniger MA, Shaffer AL, Rizzatti EG, Chapman CM (2011). Bortezomib resistance in mantle cell lymphoma is associated with plasmacytic differentiation. Blood..

[55] DeSalvo J, Kuznetsov JN, Du J, Leclerc GM, Leclerc GJ, Lampidis TJ (2012). Inhibition of Akt potentiates 2-DG-induced apoptosis via downregulation of UPR in acute lymphoblastic leukemia. Mol Cancer Res.

[56] Huiting LN, Samaha Y, Zhang GL, Roderick JE, Li B, Anderson NM (2018). UFD1 contributes to MYC-mediated leukemia aggressiveness through suppression of the proapoptotic unfolded protein response. Leukemia.

[57] Xi H, Kurtoglu M, Lampidis TJ (2014). The wonders of 2-deoxy-D-glucose. IUBMB Life.

[58] El Mjiyad N, Caro-Maldonado A, Ramirez-Peinado S, Munoz-Pinedo C (2011). Sugar-free approaches to cancer cell killing. Oncogene.

[59] Pajak B, Siwiak E, Soltyka M, Priebe A, Zielinski R, Fokt I (2019). 2-Deoxy-d-glucose and its analogs: from diagnostic to therapeutic agents. Int J Mol Sci.

[60] Laussel C, Leon S (2020). Cellular toxicity of the metabolic inhibitor 2-deoxyglucose and associated resistance mechanisms. Biochem Pharmacol.

[61] Stein M, Lin H, Jeyamohan C, Dvorzhinski D, Gounder M, Bray K (2010). Targeting tumor metabolism with 2-deoxyglucose in patients with castrate-resistant prostate cancer and advanced malignancies. Prostate.

[62] Raez LE, Papadopoulos K, Ricart AD, Chiorean EG, Dipaola RS, Stein MN (2013). A phase I dose-escalation trial of 2-deoxy-D-glucose alone or combined with docetaxel in patients with advanced solid tumors. Cancer Chemother Pharmacol.

[63] Singh D, Banerji AK, Dwarakanath BS, Tripathi RP, Gupta JP, Mathew TL (2005). Optimizing cancer radiotherapy with 2-deoxy-d-glucose dose escalation studies in patients with glioblastoma multiforme. Strahlenther Onkol.

[64] Wang M, Kaufman RJ (2014). The impact of the endoplasmic reticulum protein-folding environment on cancer development. Nat Rev Cancer.

[65] Hebert DN, Molinari M (2007). In and out of the ER: protein folding, quality control, degradation, and related human diseases. Physiol Rev.

[66] Clarke HJ, Chambers JE, Liniker E, Marciniak SJ (2014). Endoplasmic reticulum stress in malignancy. Cancer Cell.

[67] Gandhi V, Plunkett W, Cortes JE (2014). Omacetaxine: a protein translation inhibitor for treatment of chronic myelogenous leukemia. Clin Cancer Res.

[68] Mill CP, Fiskus W, DiNardo CD, Birdwell C, Davis JA, Kadia TM (2022). Effective therapy for AML with RUNX1 mutation by cotreatment with inhibitors of protein translation and BCL2. Blood.

[69] Pemmaraju N, Lane AA, Sweet KL, Stein AS, Vasu S, Blum W (2019). Tagraxofusp in blastic plasmacytoid dendritic-cell neoplasm. N Engl J Med.

[70] Prince HM, Duvic M, Martin A, Sterry W, Assaf C, Sun Y (2010). Phase III placebo-controlled trial of denileukin diftitox for patients with cutaneous T-cell lymphoma. J Clin Oncol.

[71] Desjardins A, Randazzo D, Chandramohan V, Peters KB, Johnson MO, Threatt S (2020). Phase I trial of D2C7 immunotoxin (D2C7-IT) administered intratumorally via convection-enhanced delivery (CED) for recurrent malignant glioma (MG). J Clin Oncol.

[72] Frøysnes IS, Andersson Y, Larsen SG, Davidson B, Øien JT, Julsrud L (2021). ImmunoPeCa trial: long-term outcome following intraperitoneal MOC31PE immunotoxin treatment in colorectal peritoneal metastasis. Eur J Surg Oncol.

[73] Onda M, Beers R, Xiang L, Lee B, Weldon JE, Kreitman RJ (2011). Recombinant immunotoxin against B-cell malignancies with no immunogenicity in mice by removal of B-cell epitopes. Proc Natl Acad Sci USA.

[74] Stewart SA, Dykxhoorn DM, Palliser D, Mizuno H, Yu EY, An DS (2003). Lentivirus-delivered stable gene silencing by RNAi in primary cells. RNA.

[75] Sanjana NE, Shalem O, Zhang F (2014). Improved vectors and genome-wide libraries for CRISPR screening. Nat Methods.

[76] Labun K, Montague TG, Krause M, Torres Cleuren YN, Tjeldnes H, Valen E (2019). CHOPCHOP v3: expanding the CRISPR web toolbox beyond genome editing. Nucleic Acids Res.

[77] Walter DM, Venancio OS, Buza EL, Tobias JW, Deshpande C, Gudiel AA (2017). Systematic in vivo inactivation of chromatin-regulating enzymes identifies setd2 as a potent tumor suppressor in lung adenocarcinoma. Cancer Res.

[78] Faul F, Erdfelder E, Buchner A, Lang AG (2009). Statistical power analyses using G*Power 3.1: tests for correlation and regression analyses. Behav Res Methods.

