# Supplementary material for: Targeted inhibition of protein synthesis renders cancer cells vulnerable to apoptosis by unfolded protein response
Source: Cell Death Dis. 2023 Aug 26;14(8):561. doi: 10.1038/s41419-023-06055-w (PMC10457359; doi:10.1038/s41419-023-06055-w)
Supplement: Supplementary file 1 — Supplementary Figure Legends [file 41419_2023_6055_MOESM1_ESM.docx]

**SUPPLEMENTARY INFORMATION**

**Targeted inhibition of protein synthesis renders cancer cells vulnerable to apoptosis by unfolded protein response**

**Authors/Affiliations:**

Franziska Gsottberger^1^, Christina Meier^1^, Anna Ammon^1^, Scott Parker^2^, Kerstin Wendland^1^, Rebekka George^1^, Srdjan Petkovic^1^, Lisa Mellenthin^1^, Charlotte Emmerich^1^, Gloria Lutzny-Geier^1^, Markus Metzler^3, 4^, Andreas Mackensen^1, 4^, Vidyalakshmi Chandramohan^2^, Fabian Müller^1, 4^*

^1^Department of Internal Medicine 5, Haematology and Oncology, University Hospital of Erlangen, Friedrich-Alexander University of Erlangen-Nuremberg (FAU); Erlangen, Germany.

^2^Department of Neurosurgery, Duke University Medical Center; Durham, NC, USA.

^3^Deptartment of Pediatrics and Adolescent Medicine, University Hospital of Erlangen, Friedrich-Alexander University of Erlangen-Nuremberg (FAU); Erlangen, Germany.

^4^Bavarian Cancer Research Center (BZKF), Erlangen, Germany.

*Corresponding author. Email: fabian.mueller@uk-erlangen.de

**SUPPLEMENTARY FIGURE LEGENDS**

**Fig. S1. Effects of metabolites on** **2-DG-induced cell killing and synergy with Moxe.** (**A**) JeKo-1 and Ramos were treated with fixed 2-DG concentrations and increasing concentrations of mannose, pyruvate, or ribose for 72 hours. Viability was measured by flow cytometry. Shown are representative dose-response curves normalized to untreated control. (**B**) Fold-change of Moxe activity (inverse IC_50_) after addition of 2-DG and pyruvate (pyr.) or ribose (rib.) at indicated concentrations was normalized to Moxe alone. Each bar represents mean + SD of n=3 replicates. P-values of linear trends by RM one-way ANOVA. Fold-change of activity >1 indicates synergy according to Bliss independence.

**Fig. S2.** **BiP upregulation is blocked by arrest of protein synthesis, but UPR signaling remains active in Ramos.** (**A-C**) Ramos was either treated with indicated combinations of Moxe, 2-DG, and mannose (man.) for 16 hours. Protein levels of BiP (A; n=8) and CHOP (C; n=8) were analyzed by western blot using β-Actin as control. Cleavage of XBP1 was analyzed by RT-PCR as ratio of XBP1s to XBP1 (B; n=6). Quantification was performed by densitometric analysis. Each bar represents mean ± SD. P-values were determined by ordinary one-way ANOVA (Šídák’s test). P-values: not significant (ns): p > 0.05, **: p ≤ 0.01, ****: p ≤ 0.0001.

**Fig. S3.** **Mitochondrial cell death after induction of UPR combined with protein synthesis inhibition correlates with changes in MCL-1 and BID, but not in BCL-2 or BCL-XL.** (**A-E**) Ramos and JeKo-1 were either treated with indicated combinations of Moxe, 2-DG, and mannose (man.) for 16 hours. Protein levels of cleaved PARP (A; n=8), MCL-1 (B; n=10), BCL-2 (C; JeKo-1 n=6, Ramos n=4), BCL-XL (D; JeKo-1 n=5, Ramos n=5), and BID (E; n=6) were analyzed by western blot using β-Actin as control. Quantification was performed by densitometric analysis. Each bar represents mean ± SD. P-values were determined by ordinary one-way ANOVA (Šídák’s test). Turquoise line indicates additive effects according to Bliss independence (A, B, C). P-values: not significant (ns): p > 0.05, *: p ≤ 0.05, **: p ≤ 0.01, ***: p ≤ 0.001, ****: p ≤ 0.0001.

**Fig. S4.** **Synergy is not dependent on PERK-ATF4-CHOP nor on IRE1α kinase activity.** (**A-B**) ShRNA-mediated knock-down of PERK, ATF4, CHOP, and IRE1α was performed in JeKo-1. Knock-down was confirmed by western blot in untreated or 2-DG-treated (3 mM) cells as indicated (A) and quantified by densitometric analysis compared to cells carrying a scrambled shRNA control (scr ctrl) (B). Each bar represents the mean + SD of n=3-4 replicates. P-values were determined by one-sample t-tests. (**C**) Cells carrying knock-down of PERK, ATF4, CHOP, or IRE1α and control cells were treated with Moxe and 2-DG (0.9 mM) for 72 hours. Viability was analyzed by flow cytometry and Moxe activity (inverse IC_50_) was normalized to Moxe alone. Each bar represents the mean + SD of n=3 replicates. P-values were determined by paired t-tests (PERK, ATF4, CHOP) or by RM one-way ANOVA (Dunnett’s test). (**D**) JeKo-1 was treated with Moxe (6 ng/ml) and 2-DG (3 mM) for 16 hours. Protein levels of phospho (p)-JNK, phospho (p)-p38, and β-Actin were analyzed by western blot. A representative image of n=2 replicates is shown for each protein. (**E**) JeKo-1 was treated with Moxe combined with 0.9 mM 2-DG and 3 µM Selonsertib. After 72 hours, viability was analyzed by flow cytometry. Each bar represents the mean fold-change of Moxe activity + SD of n=2 replicates. Fold-change of activity >1 indicates synergy according to Bliss independence (C, E). P-values: not significant (ns): p > 0.05, *: p ≤ 0.05, **: p ≤ 0.01, ***: p ≤ 0.001.

**Fig. S5.** **Viability after treatment with Moxe and 2-DG.** Cell lines or patient-derived cells were treated with Moxe with or without (w/o) 2-DG at indicated doses. After 72 hours, viability was analyzed by flow cytometry. Shown are representative dose-response curves normalized to untreated control.

**Fig. S6. Viability after treatment with HB21, D2C7, or LMIT-26 and 2-DG.** Cell lines were treated with immunotoxin (HB21, D2C7, LMIT-26) with or without (w/o) 2-DG at indicated doses. After 72 hours, viability was analyzed by flow cytometry or by WST-8 assay (U-87, 898). Shown are representative dose-response curves normalized to untreated control.
