# Supplementary figures and images for: Targeted inhibition of protein synthesis renders cancer cells vulnerable to apoptosis by unfolded protein response
Source: Cell Death Dis. 2023 Aug 26;14(8):561. doi: 10.1038/s41419-023-06055-w (PMC10457359; doi:10.1038/s41419-023-06055-w)

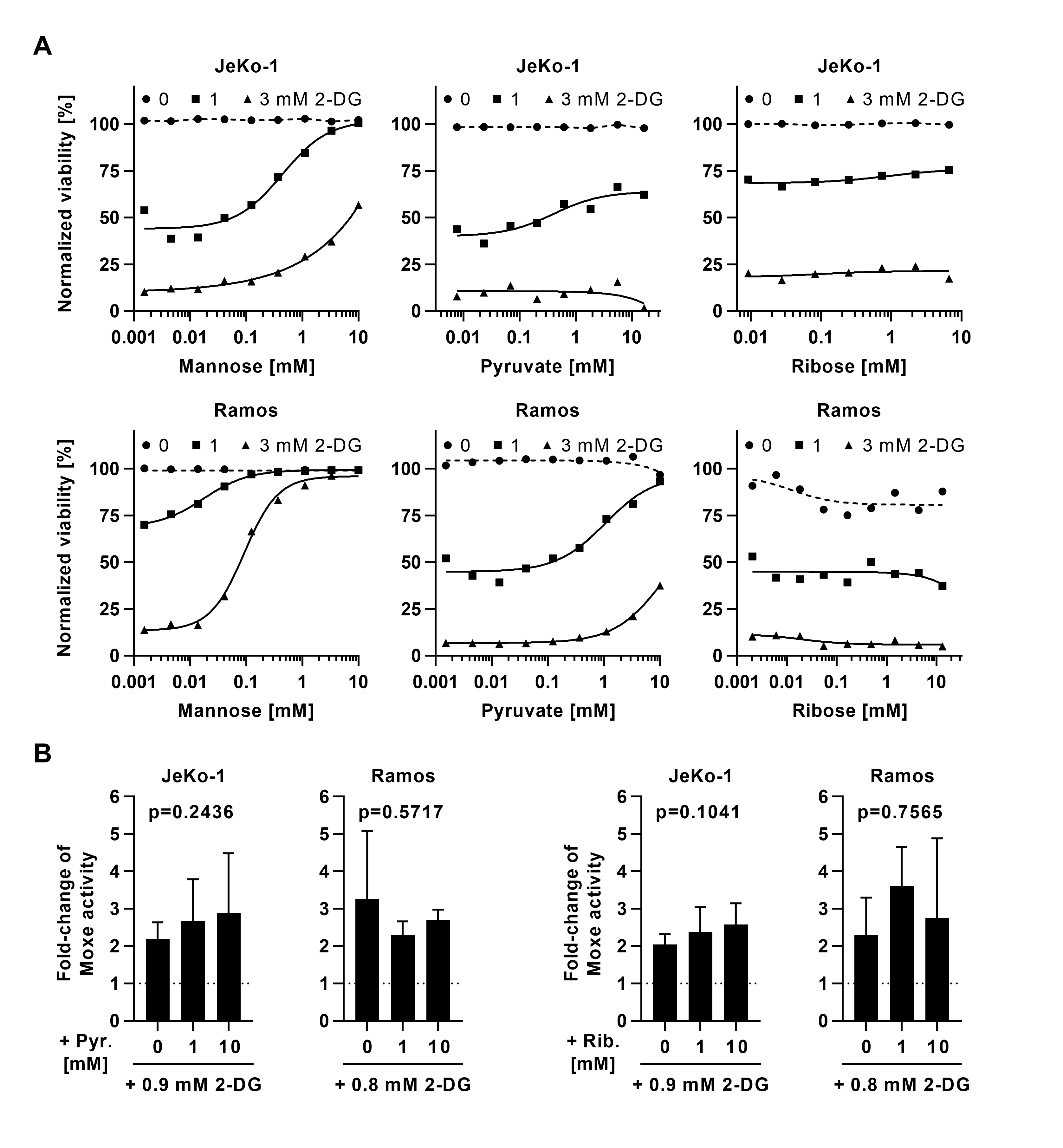

Supplement: Supplementary file 2 — Supplementary Figure S1 [file 41419_2023_6055_MOESM2_ESM.tif]

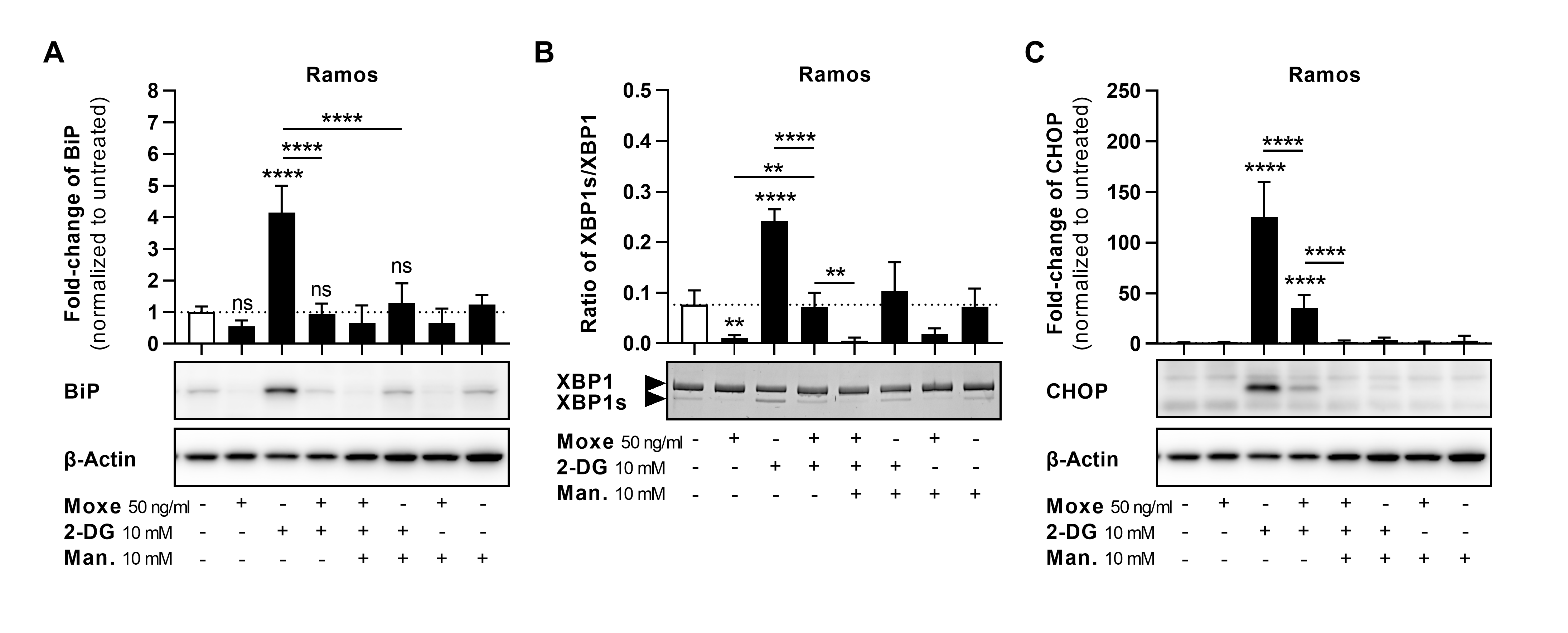

Supplement: Supplementary file 3 — Supplementary Figure S2 [file 41419_2023_6055_MOESM3_ESM.tif]

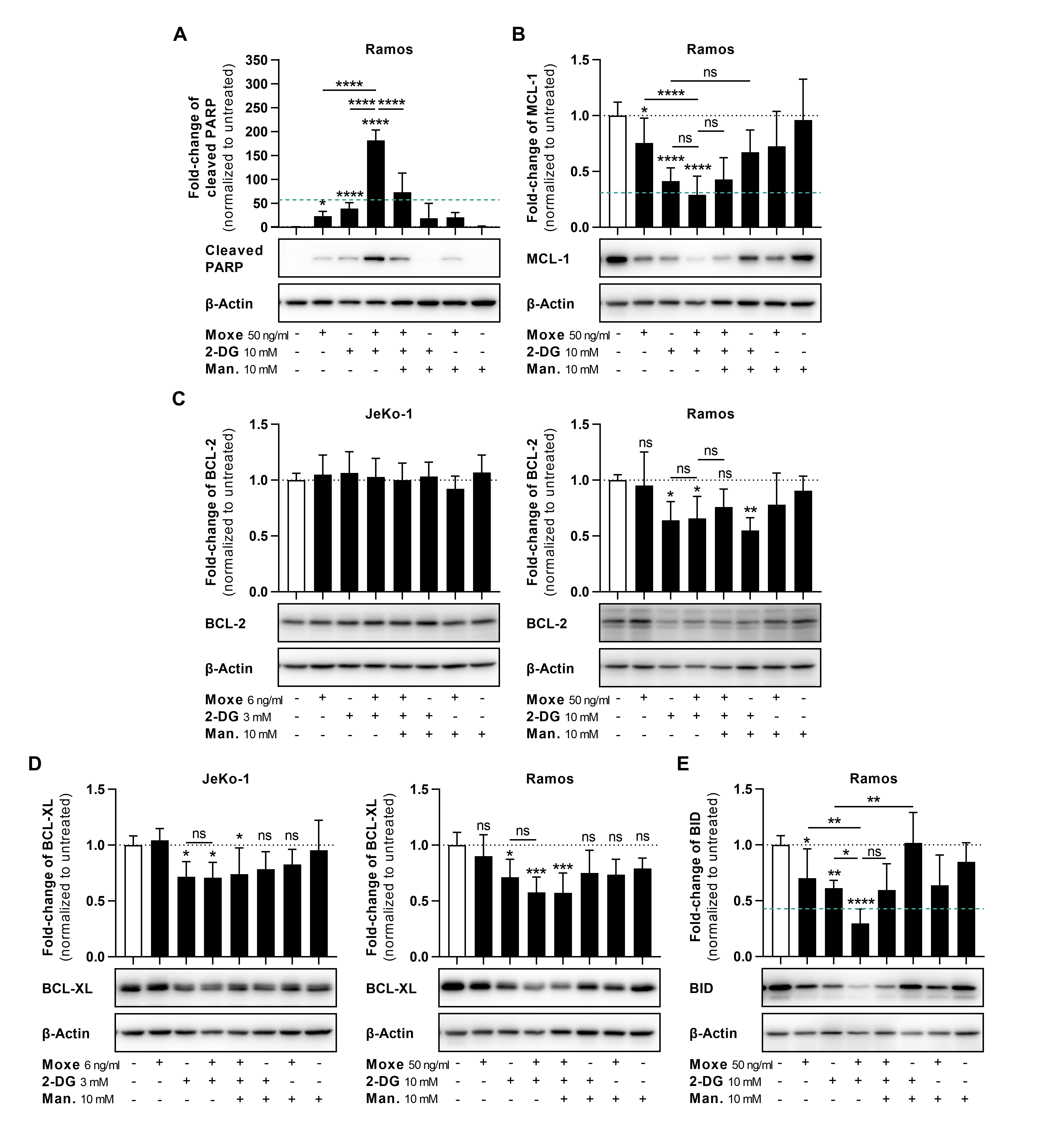

Supplement: Supplementary file 4 — Supplementary Figure S3 [file 41419_2023_6055_MOESM4_ESM.tif]

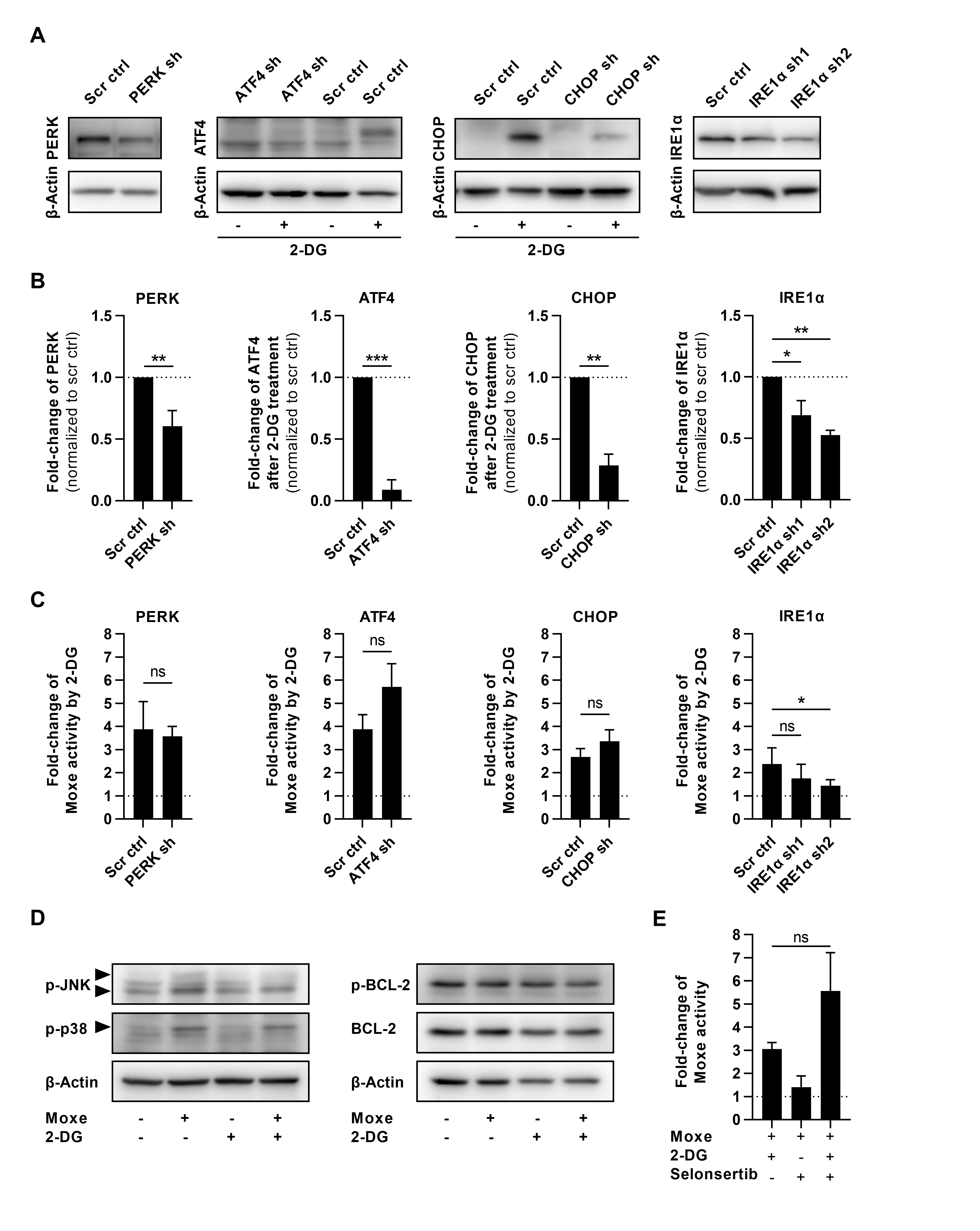

Supplement: Supplementary file 5 — Supplementary Figure S4 [file 41419_2023_6055_MOESM5_ESM.tif]

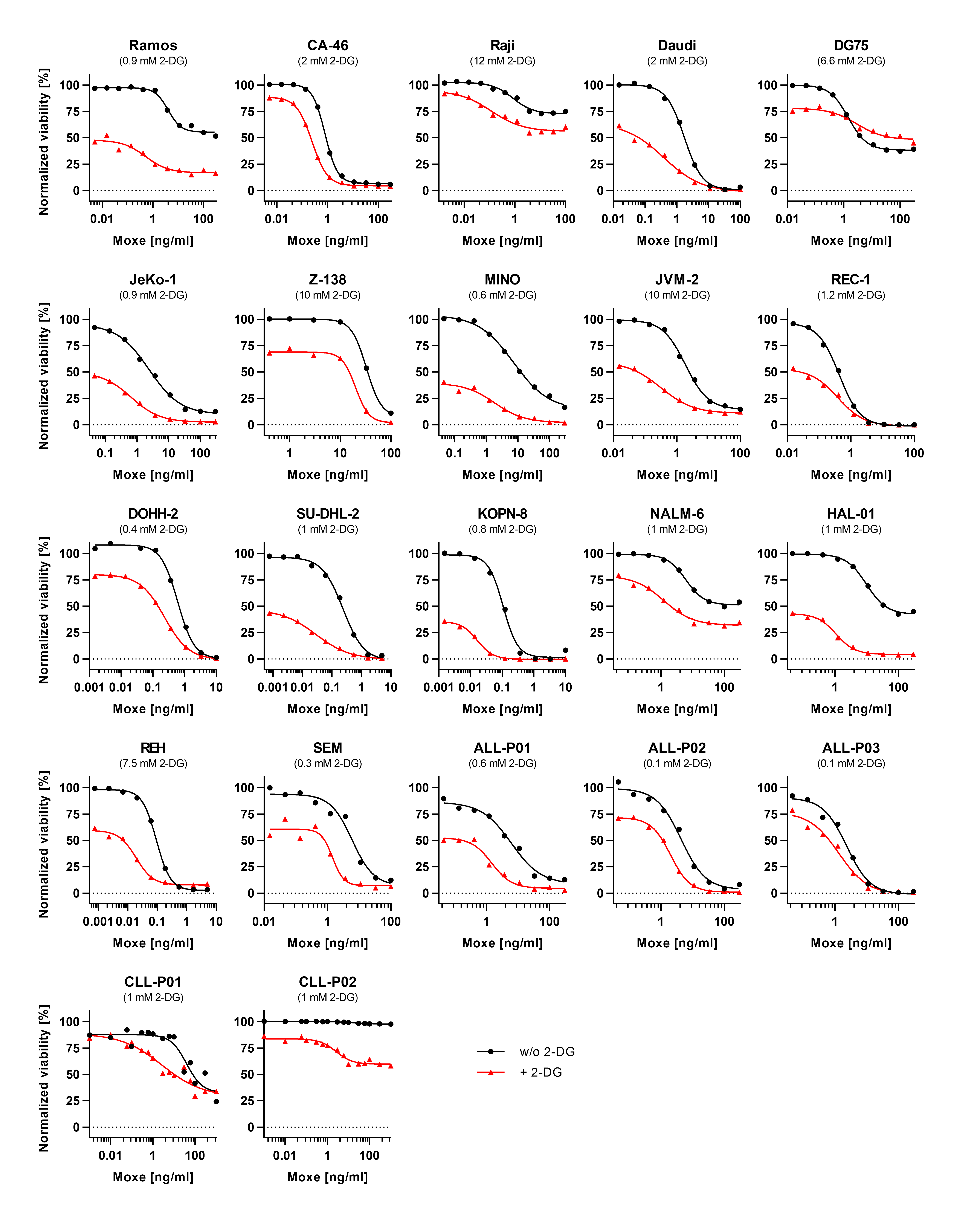

Supplement: Supplementary file 6 — Supplementary Figure S5 [file 41419_2023_6055_MOESM6_ESM.tif]

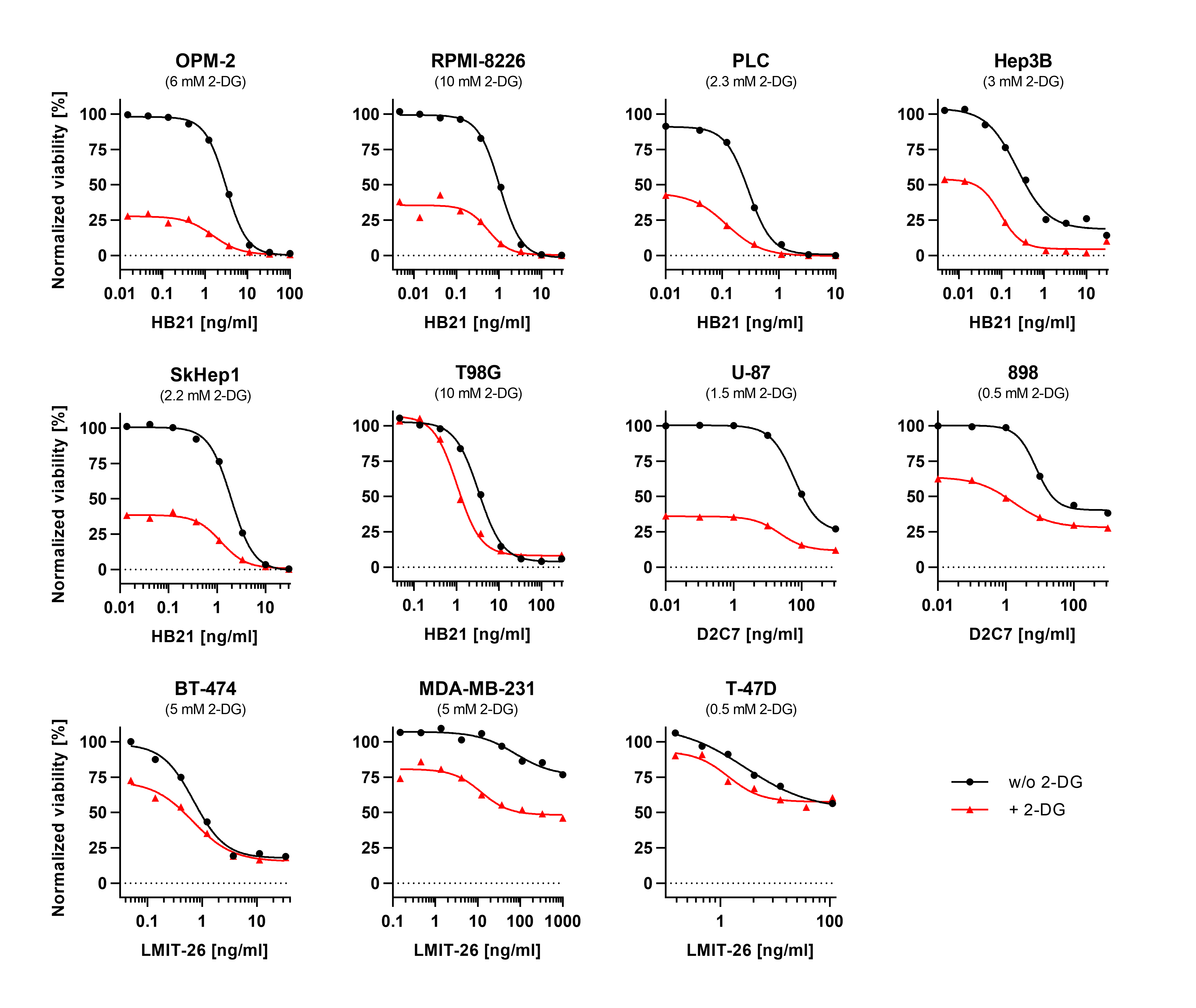

Supplement: Supplementary file 7 — Supplementary Figure S6 [file 41419_2023_6055_MOESM7_ESM.tif]
