# Supplementary material for: Targeted inhibition of protein synthesis renders cancer cells vulnerable to apoptosis by unfolded protein response
Source: Cell Death Dis. 2023 Aug 26;14(8):561. doi: 10.1038/s41419-023-06055-w (PMC10457359; doi:10.1038/s41419-023-06055-w)
Supplement: Supplementary file 11 — Original Western Blots and DNA Gels [file 41419_2023_6055_MOESM11_ESM.pdf]

SUPPLEMENTARY INFORMATION

Original Western Blots and DNA Gels

Molecular sizes are shown for each blot (kDa) and gel (bp) according to the markers used. Grey lines indicate the borders of the membrane; black lines indicate the cropped part shown in the main figures.

Figure 1A

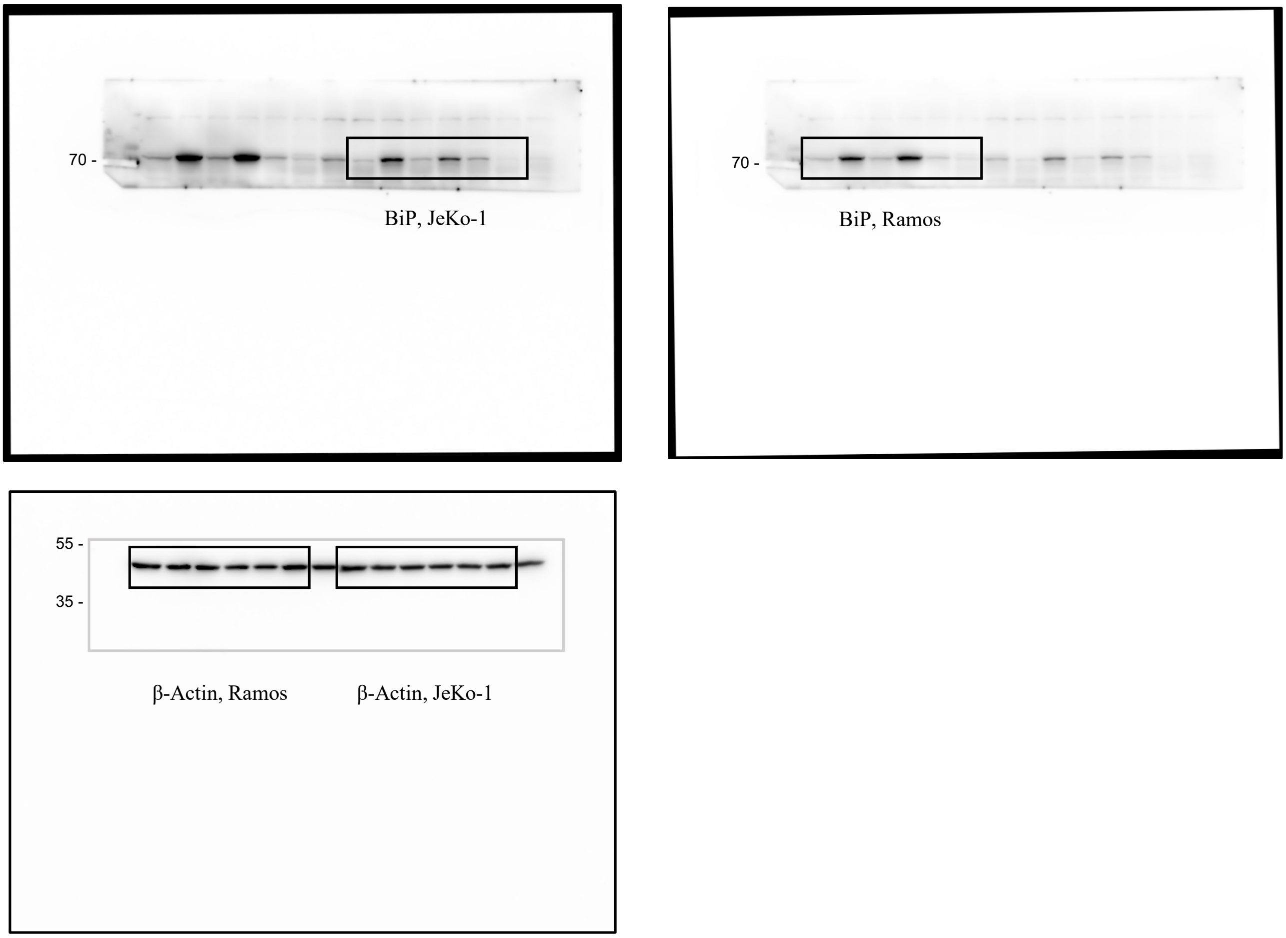

Figure 3A

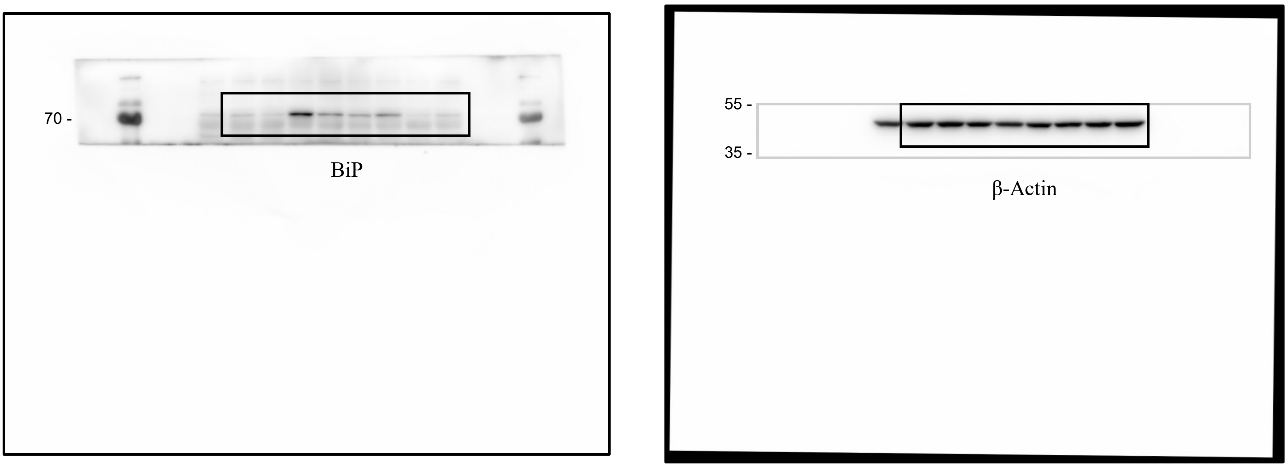

Figure 3B

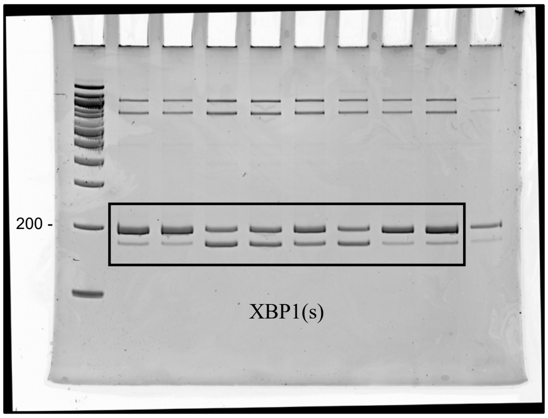

Figure 3C

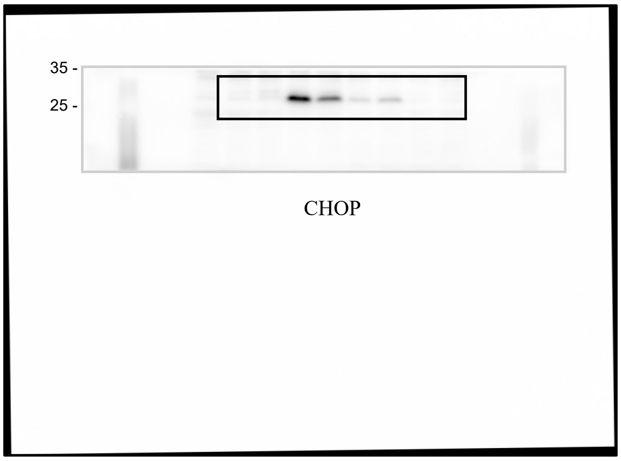

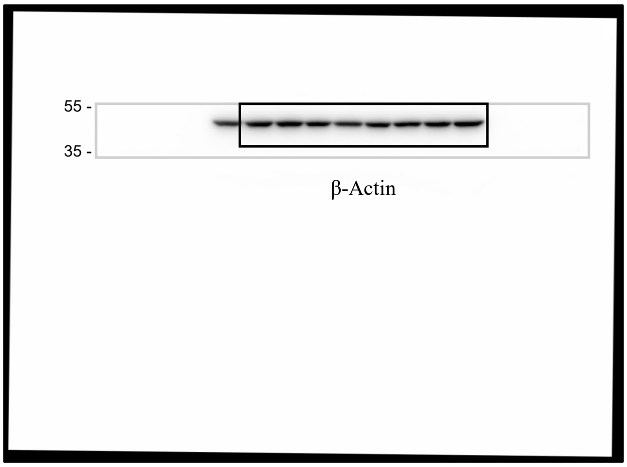

Figure 3F

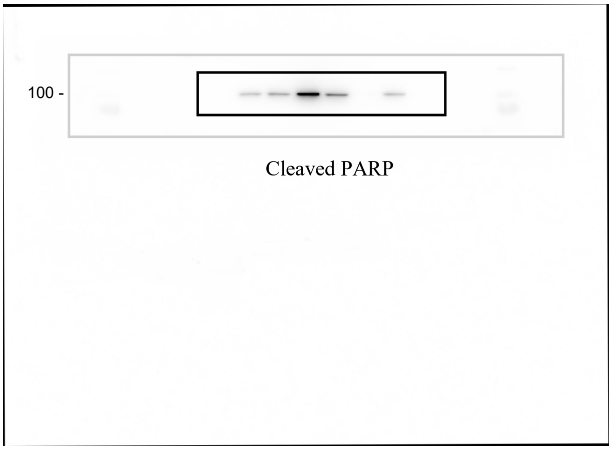

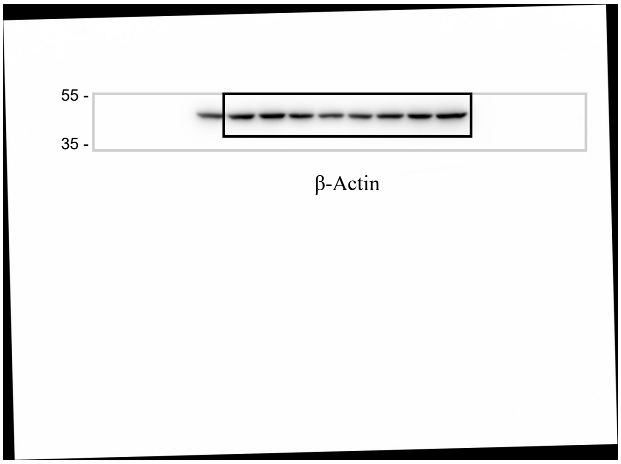

Figure 3G

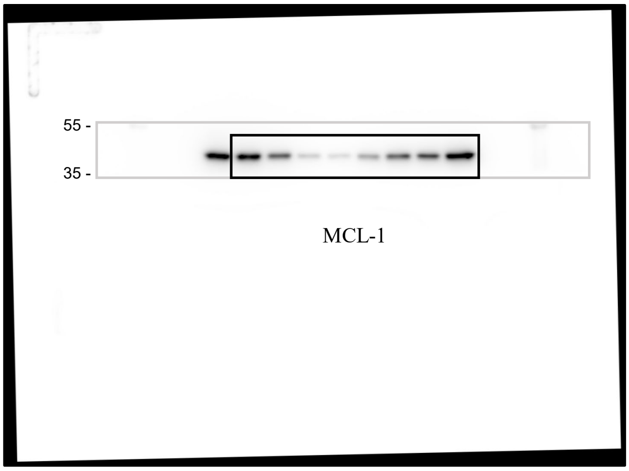

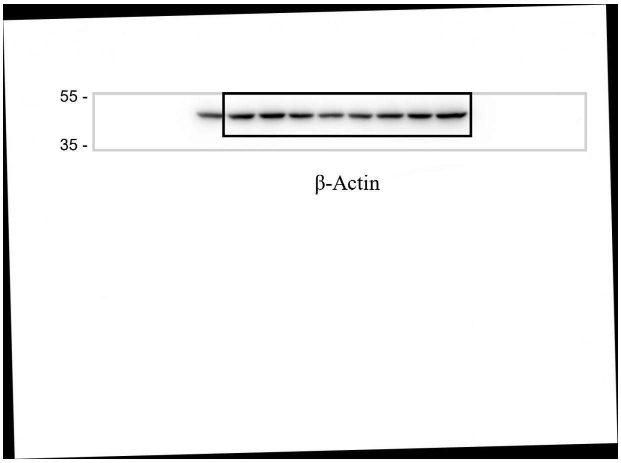

Figure 3H

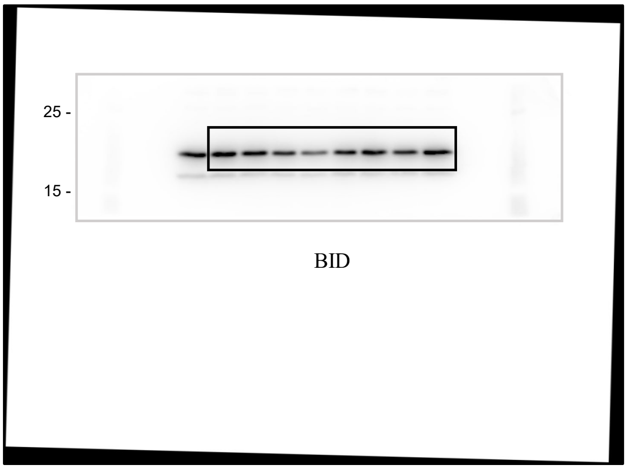

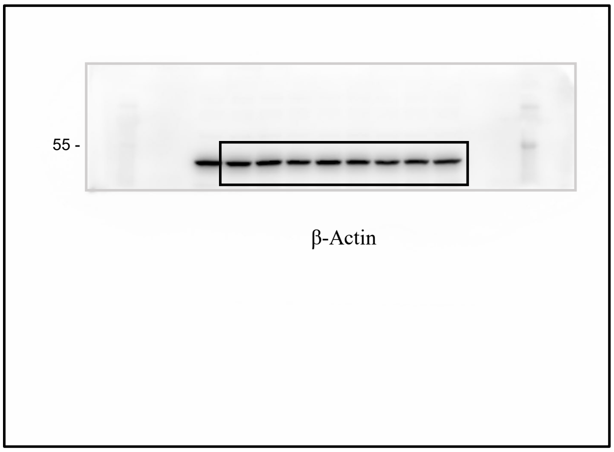

Figure 4C

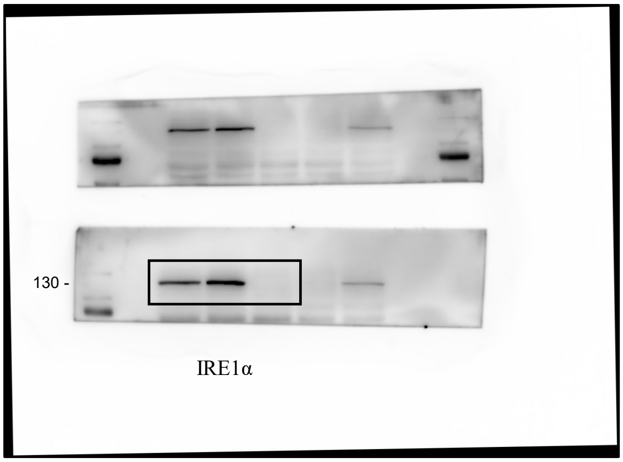

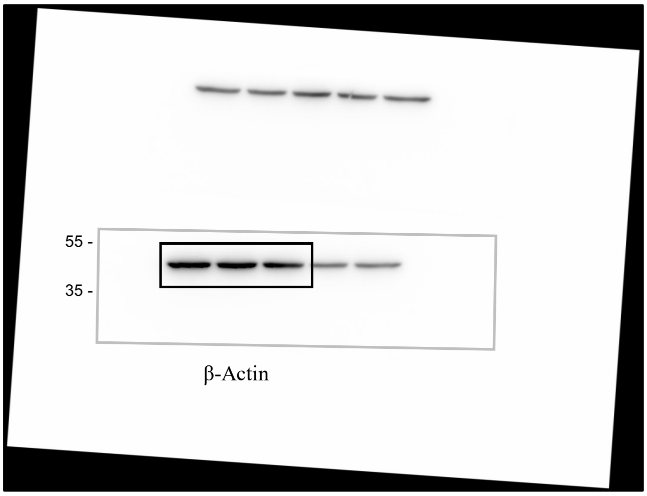

Figure 4D

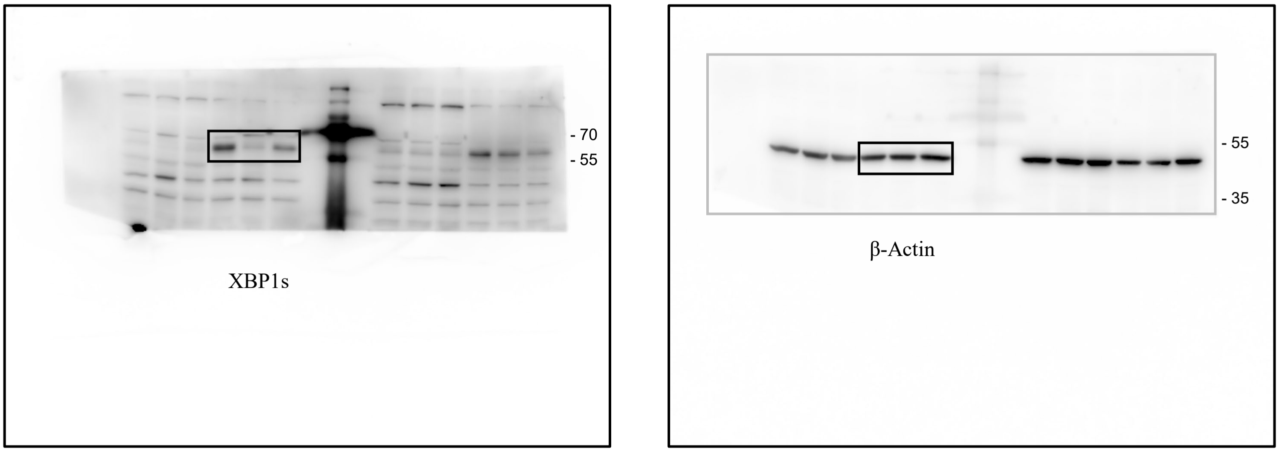

Figure 4F

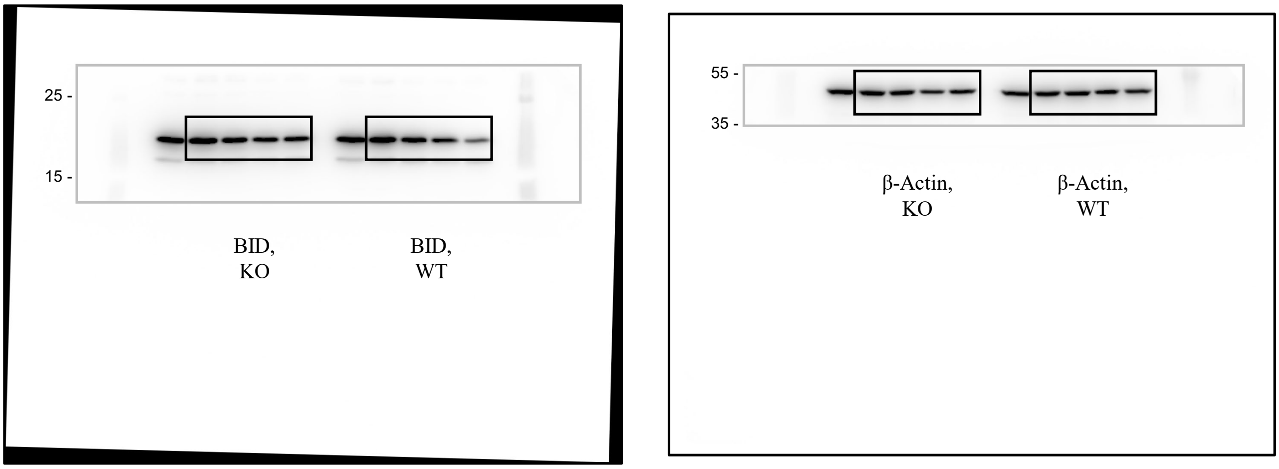

Figure 4G

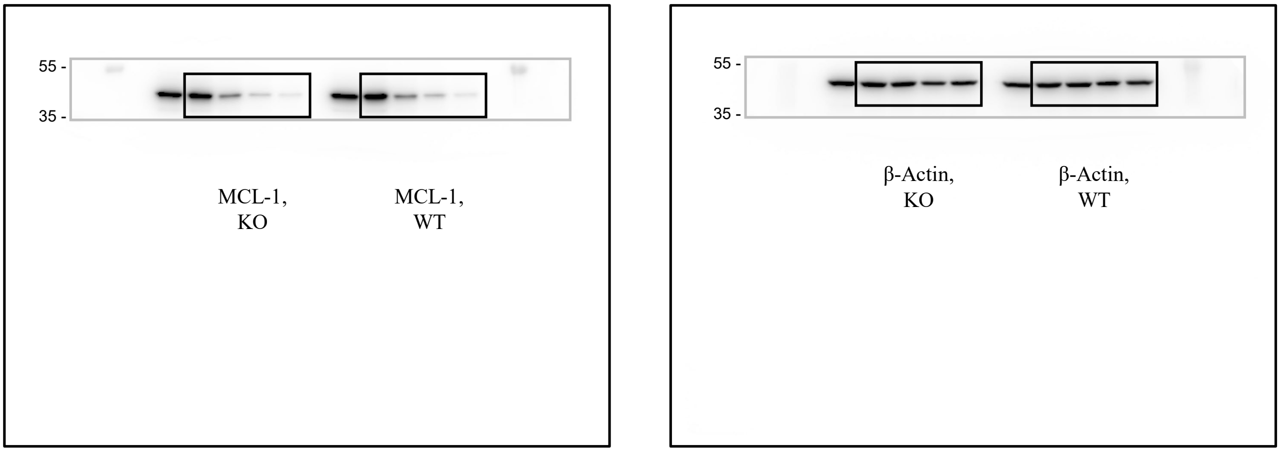

Figure 4H

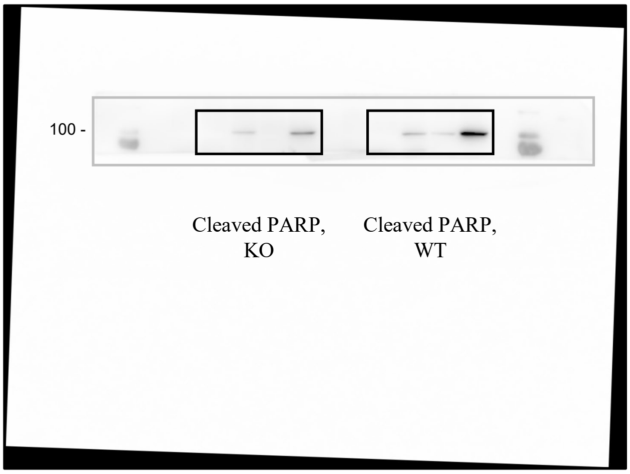

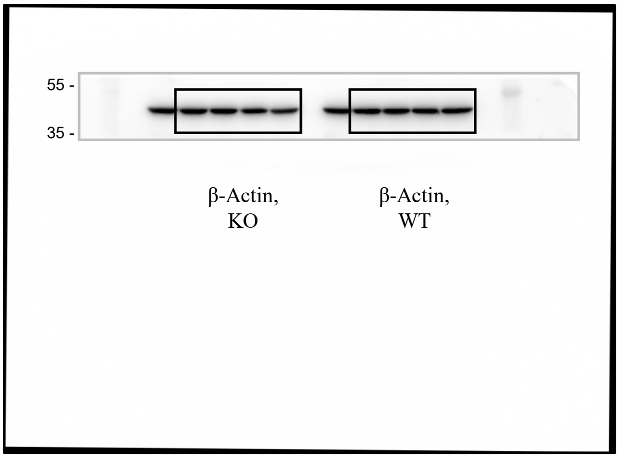

Figure 4J

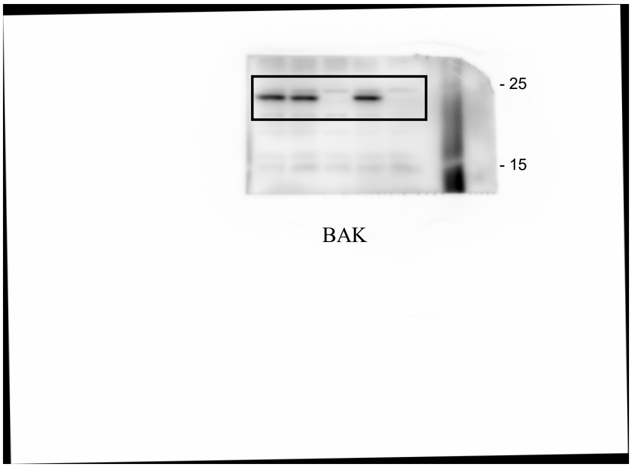

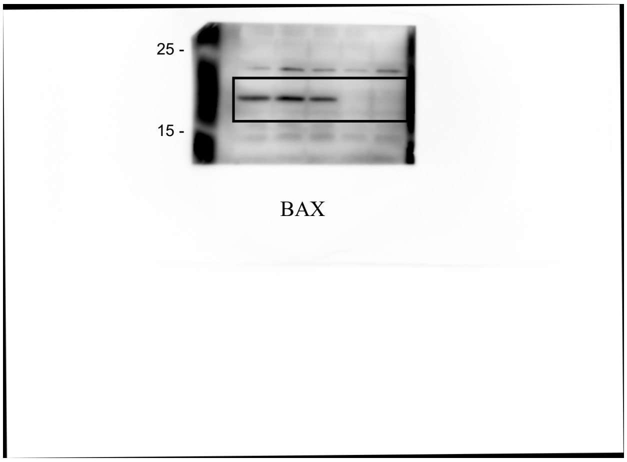

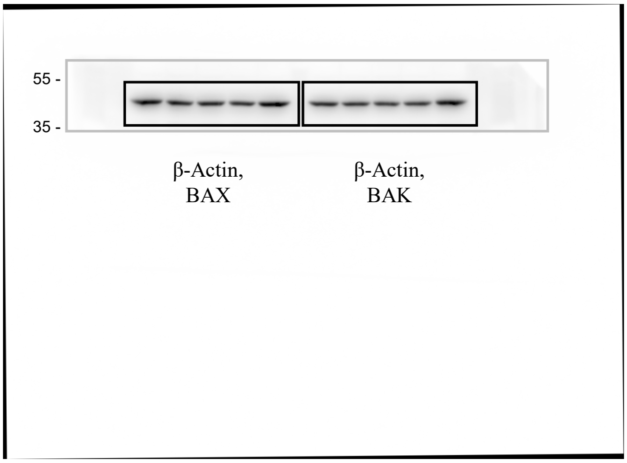

Supplementary Figure S2A

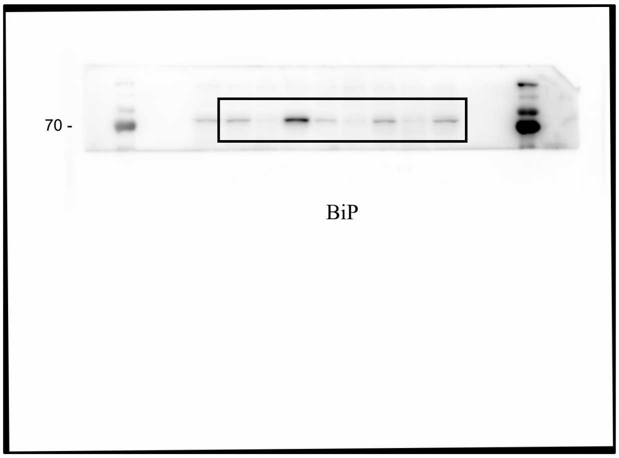

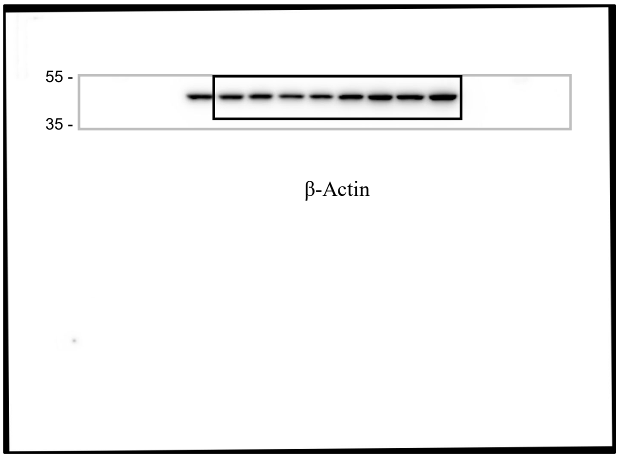

Supplementary Figure S2B

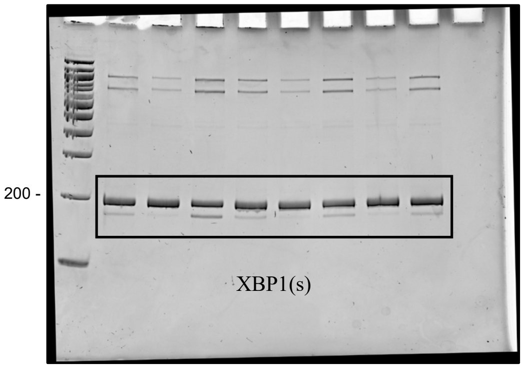

Supplementary Figure S2C

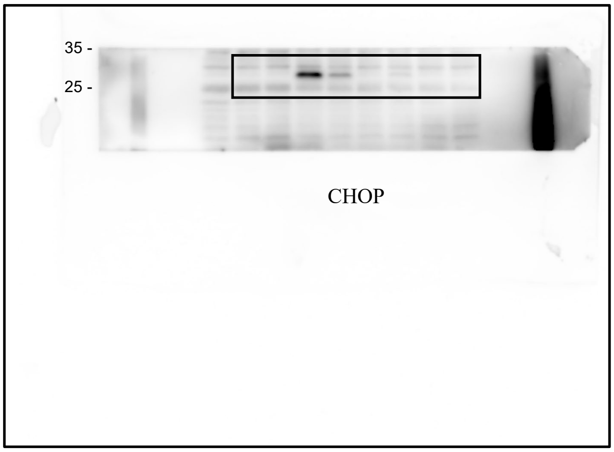

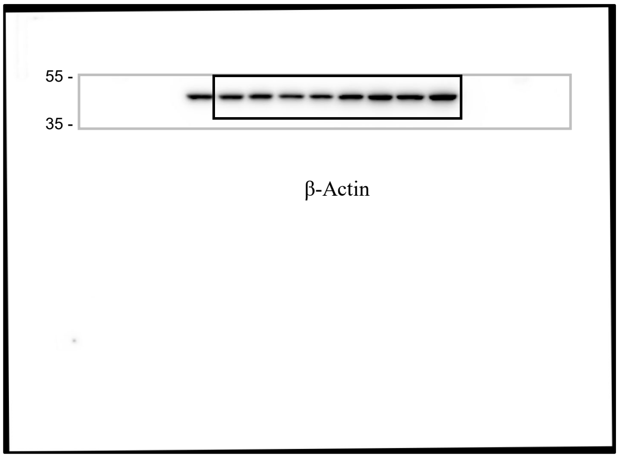

Supplementary Figure S3A

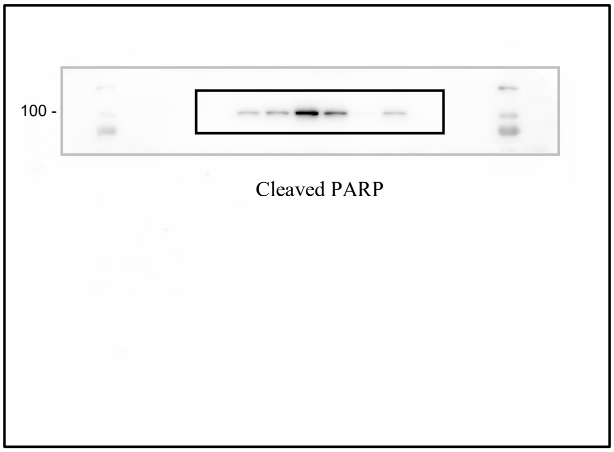

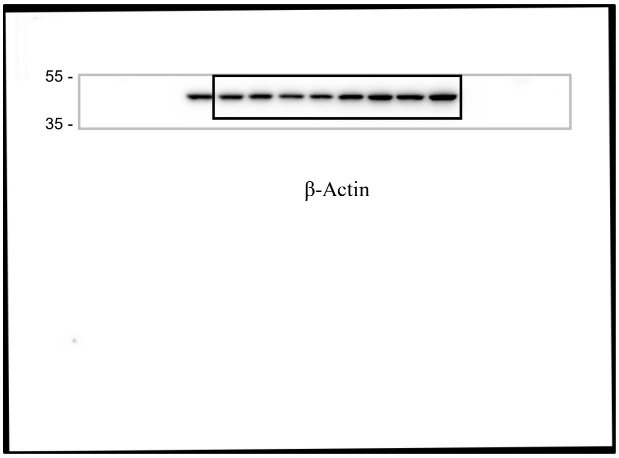

Supplementary Figure S3B

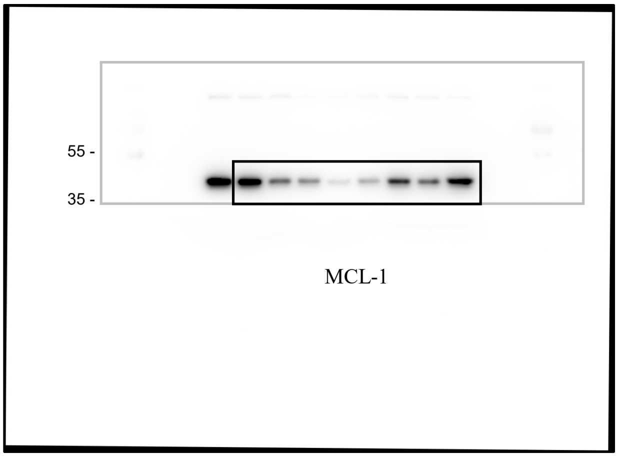

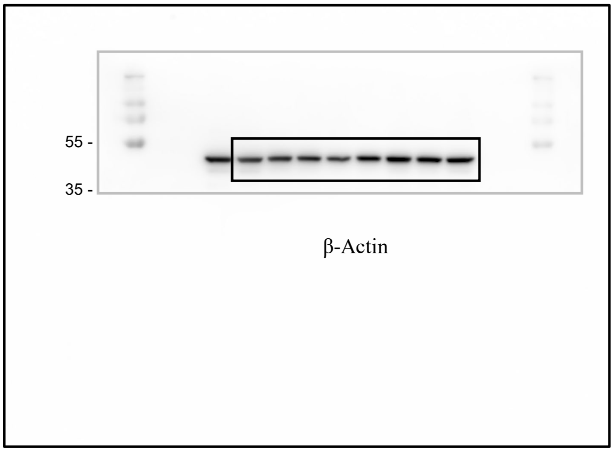

Supplementary Figure S3C

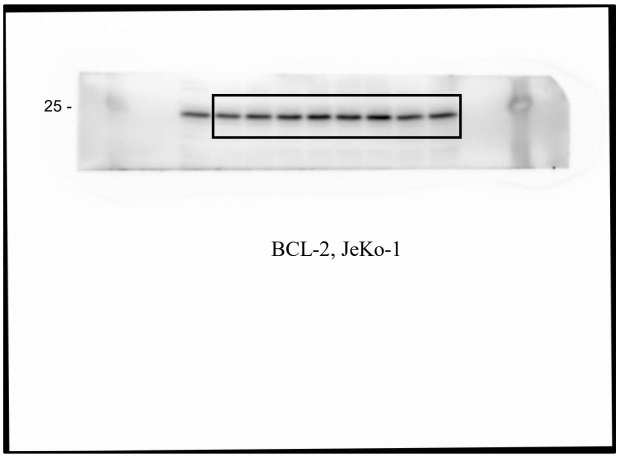

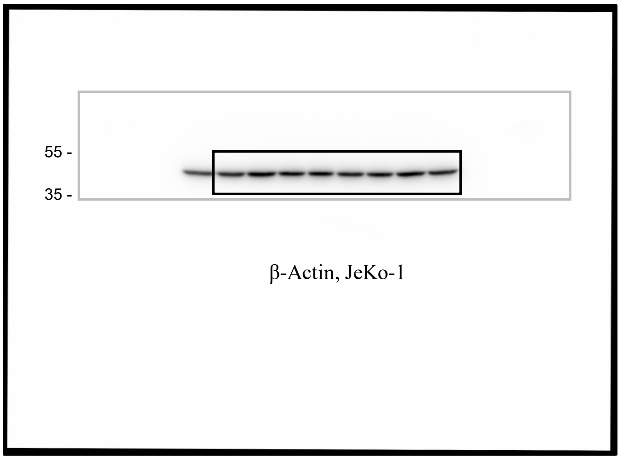

Supplementary Figure S3C

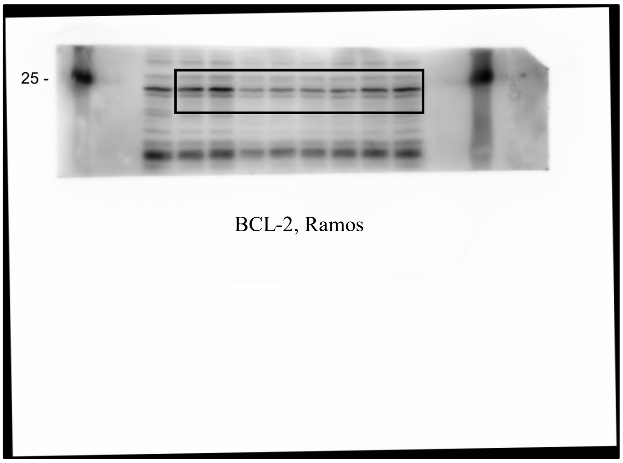

BCL-2, Ramos

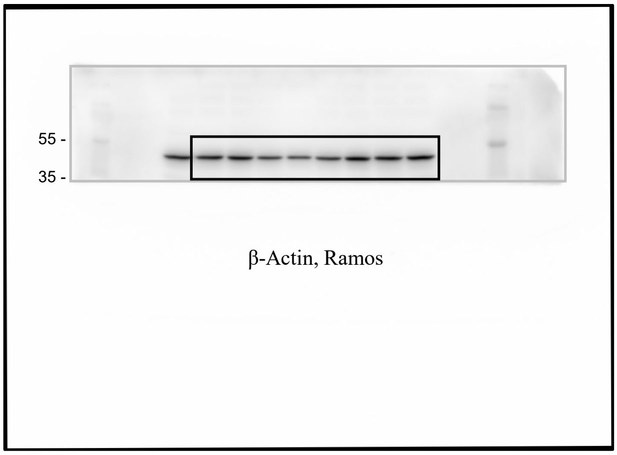

β-Actin, Ramos

Supplementary Figure S3D

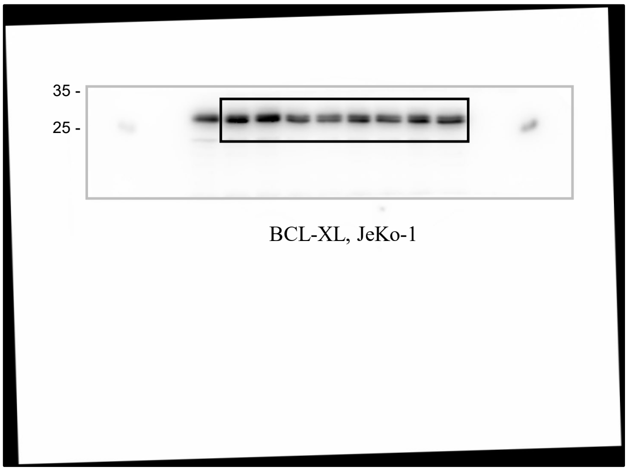

BCL-XL, JeKo-1

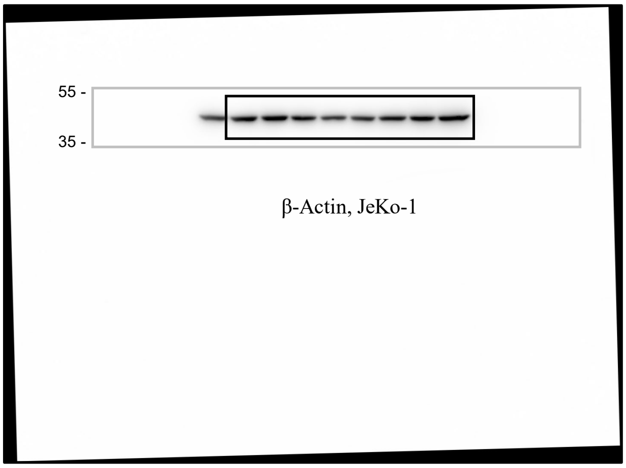

β-Actin, JeKo-1

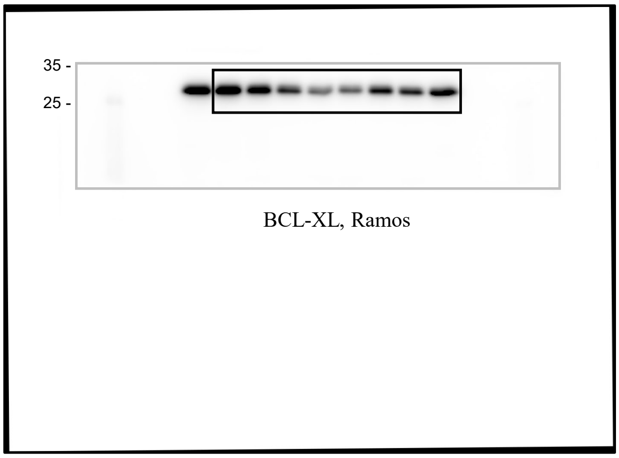

BCL-XL, Ramos

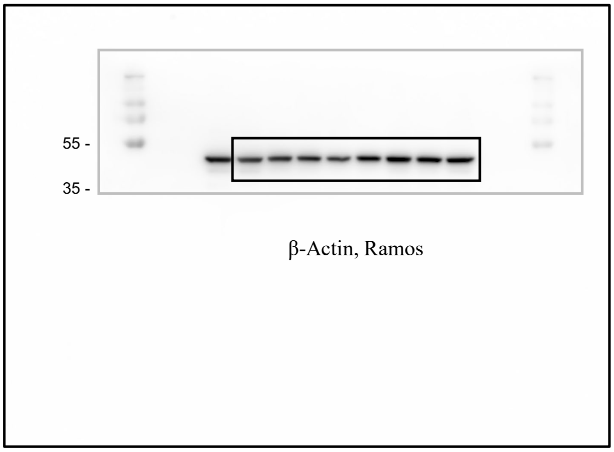

β-Actin, Ramos

Supplementary Figure S3E

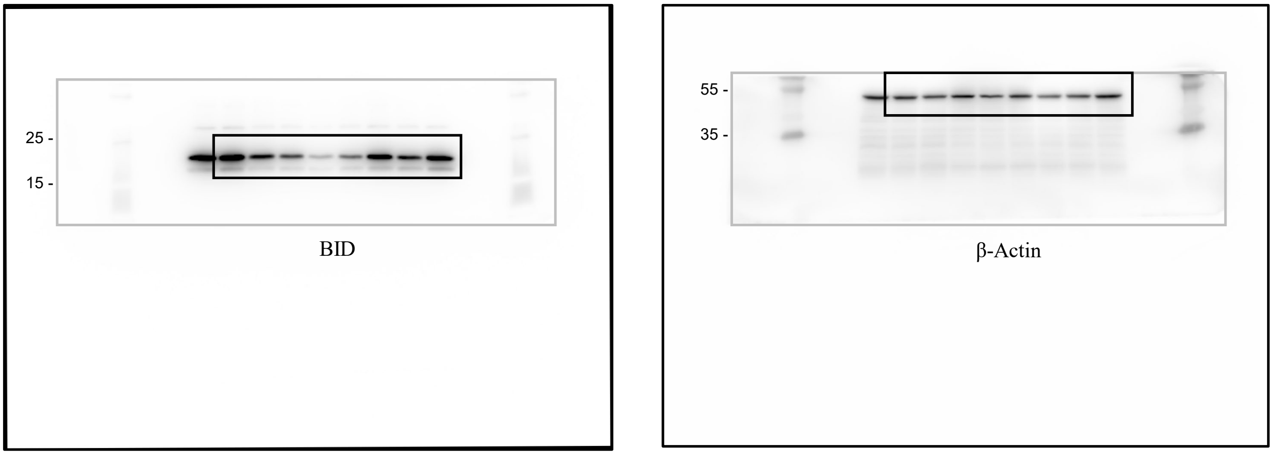

Supplementary Figure S4A

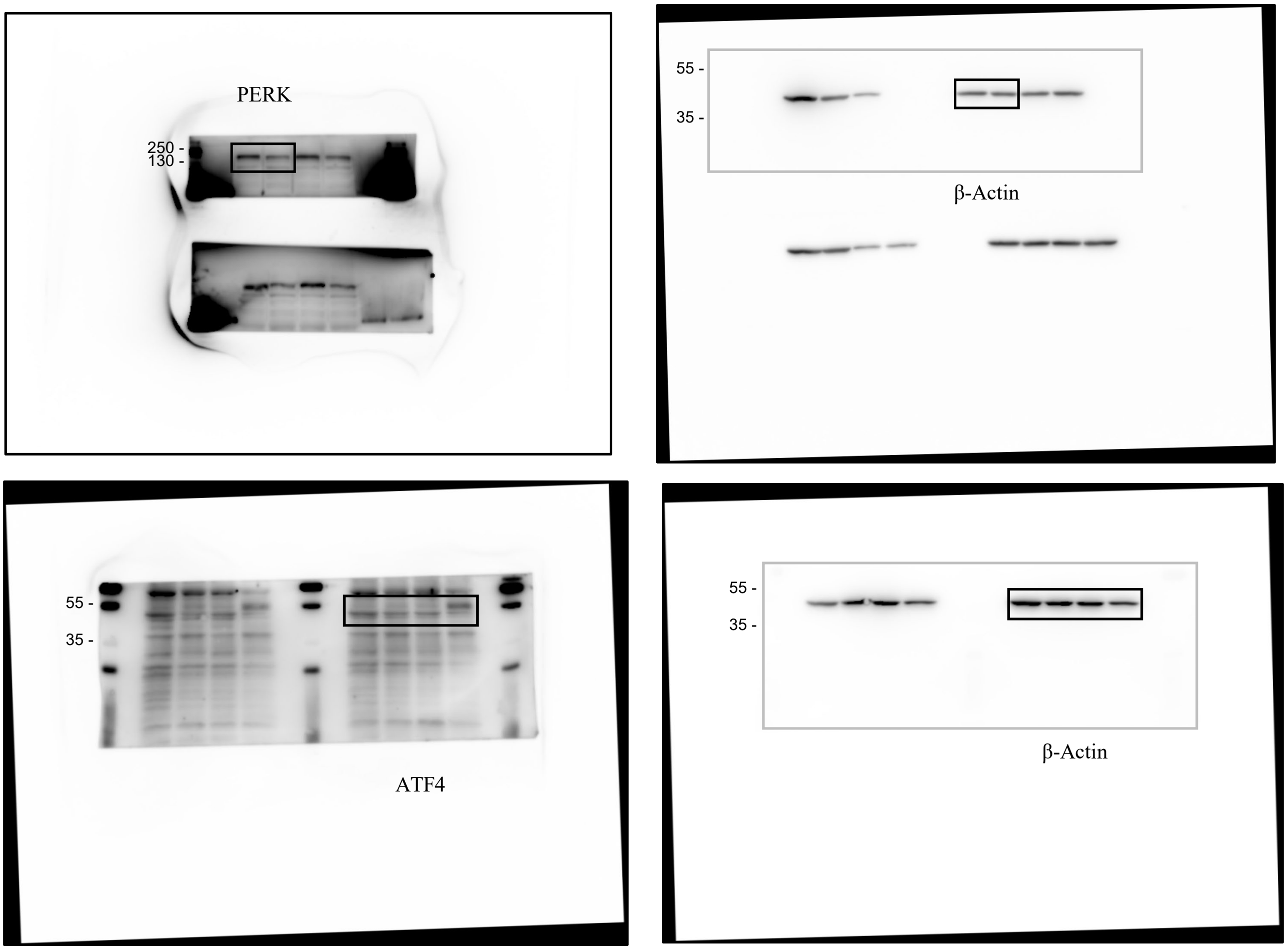

Supplementary Figure S4A

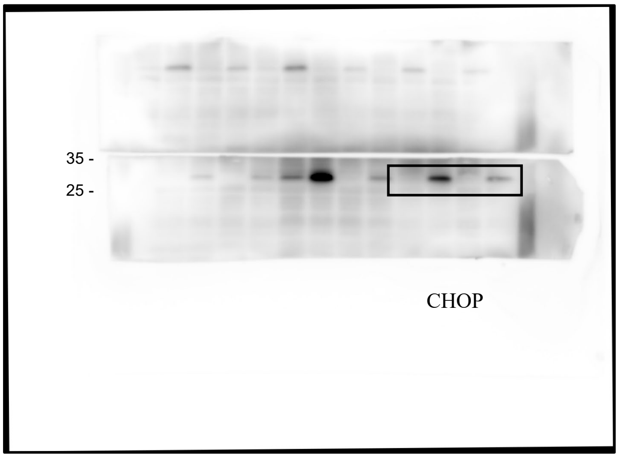

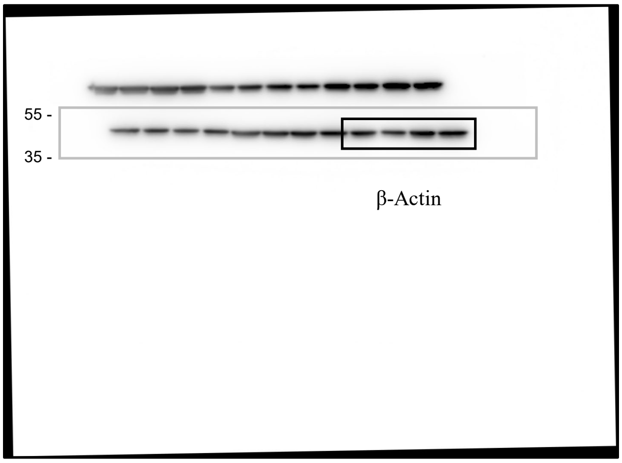

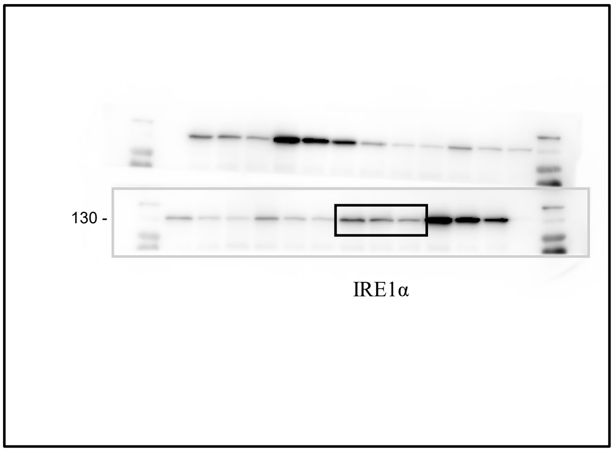

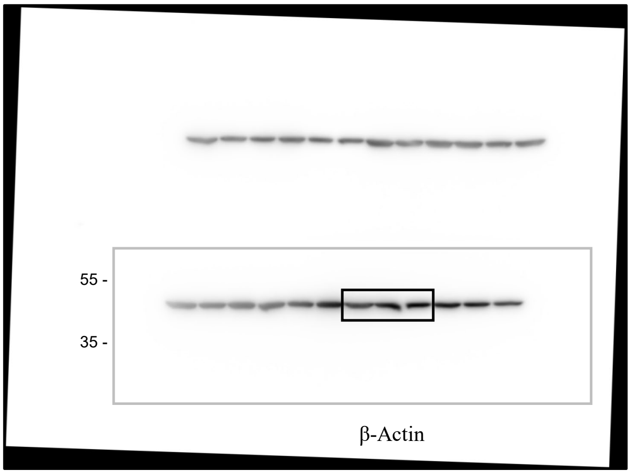

Supplementary Figure S4D

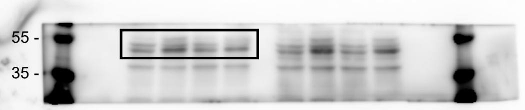

p-JNK

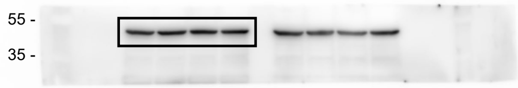

β-Actin

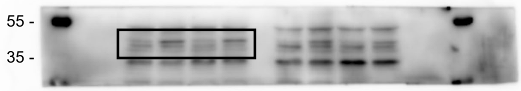

p-p38

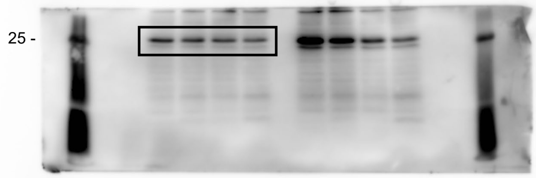

p-BCL-2

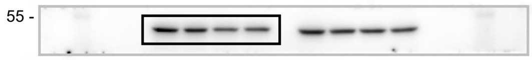

β-Actin

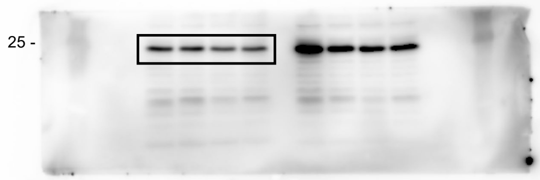

BCL-2
